# A Review of Optimization of Additively Manufactured 316/316L Stainless Steel Process Parameters, Post-Processing Strategies, and Defect Mitigation

**DOI:** 10.3390/ma18122870

**Published:** 2025-06-17

**Authors:** Usman Aziz, Marion McAfee, Ioannis Manolakis, Nick Timmons, David Tormey

**Affiliations:** 1 I-Form, the Research Ireland Centre for Advanced Manufacturing, Atlantic Technological University, F91 YW50 Sligo, Ireland; 2 Centre for Precision Engineering, Materials and Manufacturing Research (PEM Research Centre), Atlantic Technological University, F91 YW50 Sligo, Ireland; 3 Centre for Mathematical Modelling and Intelligent Systems for Health and Environment (MISHE), Atlantic Technological University, F91 YW50 Sligo, Ireland; 4 Department of Life Sciences, Atlantic Technological University, ATU Sligo, Ash Lane, F91 YW50 Sligo, Ireland; 5 WiSAR Lab, Atlantic Technological University, F92 FC93 Letterkenny, Ireland; 6 Faculty of Engineering and Technology, Atlantic Technological University, F92 FC93 Letterkenny, Ireland

**Keywords:** additive manufacturing (AM), 316/316L stainless steel, directed energy deposition (DED), selective laser melting (SLM), electron beam melting (EBM), process parameter optimization, residual stress, surface roughness, microstructure, post-processing treatments

## Abstract

The rapid progress in additive manufacturing (AM) has unlocked significant possibilities for producing 316/316L stainless steel components, particularly in industries requiring high precision, enhanced mechanical properties, and intricate geometries. However, the widespread adoption of AM—specifically Directed energy deposition (DED), selective laser melting (SLM), and electron beam melting (EBM) remains challenged by inherent process-related defects such as residual stresses, porosity, anisotropy, and surface roughness. This review critically examines these AM techniques, focusing on optimizing key manufacturing parameters, mitigating defects, and implementing effective post-processing treatments. This review highlights how process parameters including laser power, energy density, scanning strategy, layer thickness, build orientation, and preheating conditions directly affect microstructural evolution, mechanical properties, and defect formation in AM-fabricated 316/316L stainless steel. Comparative analysis reveals that SLM excels in achieving refined microstructures and high precision, although it is prone to residual stress accumulation and porosity. DED, on the other hand, offers flexibility for large-scale manufacturing but struggles with surface finish and mechanical property consistency. EBM effectively reduces thermal-induced residual stresses due to its sustained high preheating temperatures (typically maintained between 700 °C and 850 °C throughout the build process) and vacuum environment, but it faces limitations related to resolution, cost-effectiveness, and material applicability. Additionally, this review aligns AM techniques with specific defect reduction strategies, emphasizing the importance of post-processing methods such as heat treatment and hot isostatic pressing (HIP). These approaches enhance structural integrity by refining microstructure, reducing residual stresses, and minimizing porosity. By providing a comprehensive framework that connects AM techniques optimization strategies, this review serves as a valuable resource for academic and industry professionals. It underscores the necessity of process standardization and real-time monitoring to improve the reliability and consistency of AM-produced 316/316L stainless steel components. A targeted approach to these challenges will be crucial in advancing AM technologies to meet the stringent performance requirements of various high-value industrial applications.

## 1. Introduction

In recent years, significant advancements in stainless steel materials have improved its adaptability for demanding industries such as biomedical, molding, packaging, automotive, and aerospace. Researchers have examined the evolving composition and mechanical behavior of various stainless steel grades—duplex, ferritic, and austenitic—highlighting their effectiveness in diverse environments. Among these, AISI 316/316L, an austenitic stainless steel, stands out for its exceptional corrosion resistance and mechanical properties. Its composition typically includes elements such as carbon, silicon, manganese, chromium, molybdenum, nickel, phosphorus, and nitrogen, making it particularly suitable for high-performance applications in challenging environments [1,2].

Stainless steel, particularly grade 316, plays a critical role in high-precision applications due to its exceptional strength, corrosion resistance, and durability. In high-performance environments, stainless steel tools must not only exhibit superior mechanical properties but also withstand demanding conditions without compromising performance. Manufacturing processes such as injection molding, cutting, drilling, and milling involve high mechanical loads, thermal cycling, and dynamic forces, requiring materials like 316/316L stainless steel that can maintain precise dimensional tolerances and structural integrity under such demanding conditions. The overall quality of stainless steel components is heavily influenced by factors such as surface roughness, porosity, and residual stresses, all of which impact functionality and longevity. To meet these stringent requirements, both conventional manufacturing methods and additive manufacturing (AM) are employed. AM offers significant advantages, including the ability to create intricate designs and enhance material versatility. However, it also presents challenges, such as thermal stresses, surface irregularities, and residual stresses. These issues are particularly critical in 316 stainless steel, where achieving a high-quality surface finish and optimal mechanical strength is essential for reliable performance in demanding applications [3]. In contrast to traditional manufacturing, AM directly builds complex products from raw materials without relying on multiple processes like forging, casting, rolling, machining, or extrusion. AM is also more adaptable with the types of materials it uses, and leftover powder can be recycled, making it a more economical and complex shape-friendly method [4,5,6,7,8,9,10,11,12]. According to ASTM, additive manufacturing (AM) involves creating prototypes and repairing components based on specific design parameters [13]. Unlike conventional manufacturing, which depends on tools such as cutters and dies (which are often product specific and have limited lifespans), resulting in significant material waste and higher costs, AM utilizes only the required feedstock materials. This leads to more efficient material usage, reduced waste, and cost-effective production. Additionally, AM provides a high degree of design flexibility, enabling the fabrication of intricate shapes and complex structures that traditional methods struggle to achieve due to excessive material wastage and manufacturing constraints. AM techniques are categorized based on the heat source and fusion approach, including directed energy deposition (DED), powder bed fusion (PBF), material extrusion (MEX), and material jetting (MJT). Each of these methods employs different raw feedstock forms, such as wire, powder, chips, or foil, which are selected according to process requirements and intended applications [4,13,14,15]. Among these techniques, AM has gained significant recognition for its effectiveness in metallic material fabrication, with DED, selective laser melting (SLM), and electron beam melting (EBM) being the most employed methods, and Table 1 presents why these are better over other AM techniques. For this reason, this paper presents a comparative analysis of 316/316L stainless steel components produced using SLM, EBM, and DED processes [4,5,8].

In the AM of 316L stainless steel, process parameters play a crucial role in determining the mechanical properties and overall quality of the final component. For instance, in DED, laser power directly influences melt pool behavior, bonding strength, and material density, while layer height affects surface smoothness, structural uniformity, and heat distribution. Optimizing these parameters is essential for enhancing component performance and achieving superior results [16]. Similarly, in SLM, energy density is a critical factor in achieving high part density and minimizing defects. Key parameters such as hatch distance, laser power, scanning speed, and rotation angle significantly influence melt pool overlap and structural integrity [17,18]. While DED, EBM, and SLM provide substantial flexibility in creating complex geometries and minimizing material waste, they also introduce challenges that can impact the overall performance of the manufactured parts. Issues such as surface roughness, porosity, and residual stresses can degrade mechanical properties, making it essential to fine-tune AM-specific parameters such as layer thickness, laser power, scanning speed, and build orientation to ensure high-quality outcomes [19]. The impact of these parameters on additively manufactured stainless steel is significant. Poor control over layer deposition and cooling rates can lead to uneven surfaces, internal voids, and stress concentrations, all of which compromise the durability and fatigue resistance of the final component. These imperfections are particularly concerning in applications demanding high precision and structural reliability, as they increase susceptibility to fractures and mechanical failure. Therefore, understanding and optimizing AM process parameters is crucial to enhancing the performance and longevity of 316L stainless steel components in advanced engineering applications [19,20,21,22].

The primary objective of this study is to analyze the impact of additive manufacturing (AM) process parameters on the properties of 316/316L stainless steel. It critically examines the influence of specific processing parameters in direct energy deposition (DED), selective laser melting (SLM), and electron beam melting (EBM) on mechanical properties. Additionally, this review explores post-processing techniques and investigates defects such as porosity, surface roughness, and residual stresses, which collectively shape the final characteristics of stainless steel components [23]. Addressing these issues involves post-processing treatments and surface treatments to enhance wear resistance, corrosion resistance, and surface smoothness. Post-processing, particularly heat treatments, can mitigate AM’s adverse effects on stainless steel by refining microstructure, reducing residual stresses, and improving surface quality. Treatments like annealing, tempering, and solution treatment can restore strength, enhance ductility, and smooth surface irregularities, making the material more suitable for demanding applications. Thus, while AM opens new possibilities for stainless steel manufacturing, careful control of process parameters and effective post-processing steps are essential to achieving the desired properties and ensuring long-term performance [24,25,26].

In addition to parameter optimization and control from the academic literature, industry developments and patents are playing an increasingly important role in advancing additive manufacturing (AM) for 316/316L stainless steel. For instance, patented systems such as US10596626B2 detail enhanced laser-based AM systems designed to optimize melt pool characteristics and improve microstructural uniformity during processing [27]. These technologies underscore how industrial innovation complements academic optimization studies. Furthermore, process management strategies outlined in US20230211561A1 propose digital control methods to fine-tune mechanical anisotropy, ensuring more predictable performance in critical applications [28]. These contributions provide practical, real-world insight into defect mitigation and parameter selection beyond laboratory settings.

**Table 1:** Comparative analysis of additive manufacturing processes by advantages, disadvantages (including material limitations), and industrial preference [29,30,31].

AM Process	Advantages	Disadvantages	Why DED, SLM, and EBM Are Often Preferred
Directed Energy Deposition (DED)	Effective for part repair and remanufacturing Supports large builds with wire or powder feed Compatible with hybrid manufacturing setups	Rough surface finish and low precision Limited to metallic materials (e.g., titanium, Inconel) Susceptible to oxidation and porosity	Suited for aerospace and energy industries Ideal for restoring worn components and building large functional metal parts
Selective Laser Melting	Produces fully dense, high-strength metal parts Excellent detail and complex geometry capability Widely used and industrially validated	Requires high-purity fine metal powders Expensive equipment and slow build rates Needs support removal and post-processing	Essential in dental, orthopedic, and aerospace sectors for precision components with tight tolerances
Electron Beam Melting (EBM)	Excellent for titanium and nickel superalloys Reduced residual stress due to vacuum environment Ideal for creating porous implants	Limited to conductive materials Fewer materials than SLM Rough surface finish and costly setup	Preferred in orthopedic implant production and titanium aerospace parts requiring durability and lightweight structures
Fused Deposition Modeling (FDM)	Inexpensive and easy to use Good for plastic prototypes and basic fixtures Supports a wide range of thermoplastics	Poor surface finish and accuracy Limited to polymers (PLA, ABS, etc.) Weak mechanical and thermal properties	Not applicable for high-strength metal parts; primarily a prototyping tool
Vat Photopolymerization (SLA/DLP)	High-resolution surface finish Excellent for detailed models and dental molds Efficient for visual prototypes	Restricted to photopolymer resins Brittle and not load-bearing Requires post-curing and careful handling	Lacks durability and material versatility for functional or structural components
Binder Jetting	Fast build speed No support structures needed Works with metals, ceramics, and sand	Requires sintering or infiltration post-processing Results in lower-density parts Poor for high-stress applications	Less suitable for aerospace and medical implants due to part strength concerns
Material Jetting	High detail and multi-material printing Full color capability Smooth surface finishes	Fragile parts and high material cost Limited to resins with low thermal/mechanical strength	Primarily used for visual or anatomical models, not structural parts
Sheet Lamination	Cost-effective and low waste High-speed for paper or plastic models	Weak bonding and mechanical strength Limited metal use in ultrasonic systems Not suitable for complex geometries	Not used for load-bearing or precision metal applications

In contrast to broader reviews on metal additive manufacturing, this paper emphasizes the use of specific AM techniques—DED, SLM, and EBM—for processing 316/316L stainless steel, providing a targeted examination of their capabilities and challenges. It evaluates the relationship between process parameters, defect formation, and post-processing strategies, assessing their combined impact on microstructures, and mechanical properties in high-performance engineering applications. The key focus areas include the following:

- Optimization and influence of process parameters: We investigate how AM process factors—such as laser power, scanning speed, layer thickness, etc.—affect mechanical attributes like hardness, tensile strength, and fatigue resistance. This study further examines parameter optimization and evaluates the suitability of each AM technique based on its application-specific strengths and constraints.
- Defects and their consequences: We identify typical defects in AM-produced 316/316L stainless steel, including residual stresses, porosity, and surface inconsistencies. These issues can weaken material integrity, necessitating targeted mitigation strategies to meet rigorous engineering requirements.
- Post-processing methods: We explore post-processing techniques such as heat treatment, hot isostatic pressing (HIP), and stress-relief processes, which are essential for improving microstructure, enhancing mechanical properties, and minimizing defects. These treatments contribute to superior dimensional accuracy and surface finish, making AM-fabricated stainless steel viable for applications requiring high precision and reliability.

By establishing this interconnection between process control, defect management, and post-processing optimization, this study provides a comprehensive framework for advancing AM techniques in critical engineering sectors such as aerospace, biomedical, and automotive industries.

## 2. DED, SLM, and EBM Process Parameter Effects and Optimization

Before proceeding with in-depth research, it is essential to understand the reasons why DED, SLM, and EBM are considered superior to other standard additive manufacturing (AM) processes. Table 1 outlines a detailed comparison highlighting their advantages, material limitations, and industrial relevance.

The mechanical properties of 316/316L stainless steel are highly influenced by the selected additive manufacturing (AM) technique and its process parameters. This section compares selective laser melting (SLM), Directed energy deposition (DED), and electron beam melting (EBM), emphasizing the distinct characteristics of each method. It provides a detailed assessment of how their unique operational parameters shape the microstructure and overall performance of stainless steel alloys, offering valuable insights into their suitability for various applications [16]. This comparative analysis highlights key differences in process conditions, powder delivery methods, and preheating requirements, while also addressing defect mitigation through post-processing strategies—factors that play a crucial role in determining the final material properties. Each of the AM techniques operates differently, utilizing distinct energy sources—DED and SLM rely on lasers, while EBM uses an electron beam. Their power capacities also vary significantly, with DED operating at around 500 W, SLM at a lower 120 W, and EBM reaching up to 3500 W. Additionally, the beam sizes differ across these methods, affecting precision and material deposition. DED has a broader beam range of 660 to 900 µm, SLM features a much finer beam of 30 to 250 µm, and EBM falls between 200 and 1000 µm. Another key difference lies in preheating requirements, which play a crucial role in material properties and defect mitigation. DED requires the build plate to be heated between 200 °C and 500 °C, whereas SLM operates at lower preheating temperatures of around 100–200 °C. In contrast, EBM demands significantly higher preheating, typically around 700 °C, which helps in reducing residual stresses. Scanning speed also varies among these techniques—DED operates at a slower rate of 0.001 to 0.04 m/s, while SLM moves at a moderate pace of 0.3 to 1 m/s. EBM, however, achieves much higher speeds, often exceeding 1000 m/s, making it ideal for applications requiring rapid production. The thickness of each deposited layer further differentiates these processes. DED builds thicker layers ranging from 200 to 1000 µm, whereas SLM achieves finer layer deposition between 20 and 100 µm, and EBM falls in between, with layer thicknesses from 50 to 200 µm [32,33,34,35,36,37,38,39,40].

Post-processing is an essential step in ensuring the final product meets quality and performance standards. In DED, post-processing requirements vary based on application, as stress relief may be necessary in some cases. SLM, on the other hand, generally requires post-processing treatments such as hot isostatic pressing (HIP) to enhance mechanical properties and eliminate residual stresses. In contrast, EBM usually requires minimal post-processing, as its high-temperature preheating helps mitigate internal stresses during fabrication. The mechanical properties of components also depend on the AM method used. Both DED and SLM tend to exhibit lower ductility but maintain or even surpass the yield strength of conventionally manufactured materials. EBM, however, produces mechanical properties comparable to traditional manufacturing techniques, offering a balance between strength and stability. Surface quality is another factor that differentiates these techniques. DED often results in rougher surfaces, with roughness values ranging from 20 to 50 µm, depending on the laser beam size. In comparison, SLM produces a much smoother finish, often below 10 µm, making it ideal for precision applications. EBM falls in between, with surface roughness ranging from 10 to 50 µm. Additionally, residual stress accumulation varies among these methods. Both DED and SLM generate high residual stresses due to rapid thermal cycles, often necessitating post-processing for stress relief. In contrast, EBM minimizes residual stress formation thanks to its higher preheating temperatures, which create a more uniform thermal distribution throughout the build. These differences in processing techniques make each AM method suitable for specific applications and materials. DED is widely used in aerospace, medical, dental, tooling, and high-tech industries for fabricating direct metal components. It is compatible with materials such as stainless steel, titanium alloys, cobalt-chromium, tool steels, nickel-based alloys, and aluminum alloys. SLM is preferred for its precision and is commonly employed in aerospace, medical, automotive, and tooling applications, working well with materials like titanium alloys (Ti, Ti6Al4V), cobalt–chromium, and nickel-based superalloys. Meanwhile, EBM is often utilized in the energy, aerospace, medical device, automotive, tooling, and defense industries, supporting materials such as tool steels, stainless steels, titanium alloys, and nickel-based alloys. By understanding the unique characteristics of each AM process, industries can select the most suitable method for their specific needs, balancing factors such as precision, mechanical properties, surface quality, and production efficiency. This comparison highlights how advancements in AM technology continue to push the boundaries of material fabrication, making it a critical solution for high-performance applications [4,34,41,42,43,44].

There are different manufacturers providing equipment for industrial and laboratory applications, such as DED manufactured by Optomec [45], SLM manufactured by Realizer GmbH [46], and EBM manufactured by Arcam EBM S12 [47]. DED, as employed by the Optomec LENS 750 system, is widely used in the aerospace and defense sectors for repairing and rebuilding high-value metal components such as turbine blades, engine parts, and structural brackets, with users including GE Aviation, NASA, and United Technologies. For instance, GE Aviation uses DED to repair aircraft engine components, and NASA has utilized LENS systems for rebuilding rocket engine nozzles. DED is also applied in the oil and gas industry for refurbishing large metal parts like valves and drill heads and in tooling/molds industries for adding material to worn molds [45]. The Realizer SLM 50, a high-precision metal printer, is especially prevalent in the medical/dental, aerospace, and automotive industries. In dental laboratories, the SLM 50 is used to manufacture custom crowns, bridges, and orthodontic devices using cobalt–chrome and titanium alloys. Airbus employs SLM technology for lightweight, topologically optimized aircraft brackets, while BMW and Bugatti use it for complex, high-performance engine parts and brake calipers. Its high resolution makes it ideal for jewelry prototyping as well [46]. The Arcam EBM S12 is a key technology in the orthopedic medical and aerospace industries. EBM is used by companies like Exactech and LimaCorporate to manufacture hip implants, spinal cages, and other porous titanium orthopedic implants, taking advantage of EBM’s ability to create controlled porosity for bone ingrowth. In aerospace, Boeing and Avio Aero (GE Aviation) use Arcam systems to print lightweight, high-strength titanium parts that withstand extreme conditions, such as engine brackets and support structures [47]. Based on this discussion, the comparative analysis of DED (Optomec 750 [45]), SLM (Realizer SLM50 [46]), and EBM (Arcam EBM S12 [47]) has been summarized in Table 2.

### 2.1. Direct Energy Deposition (DED)

The mechanical properties of 316L stainless steel are significantly influenced by build orientation during additive manufacturing. For example, components fabricated in a horizontal orientation (0-degree build angle) generally exhibit higher tensile and yield strengths compared to those produced in a vertical orientation (90-degree build angle). This difference arises because, in the 0-degree orientation, the applied load is parallel to the build layers, whereas in the 90-degree orientation, it is perpendicular. As a result, variations in interlayer bonding and material anisotropy directly impact overall mechanical performance. The cited study further investigated the mechanical behavior of 316L stainless steel fabricated at both 0-degree and 90-degree build orientations [48].

**Figure 1:**
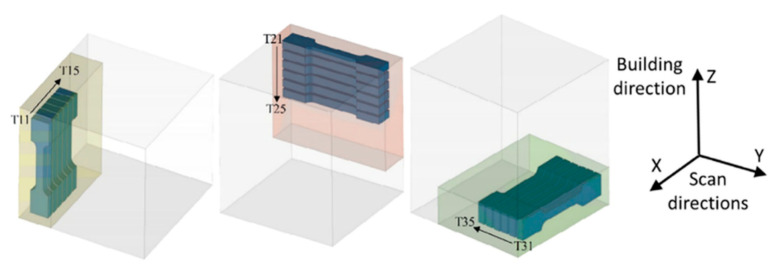
DED-processed 316L stainless steel cubic component with test specimens oriented in three different directions (sourced from publisher under permission) [48].

**Figure 2:**
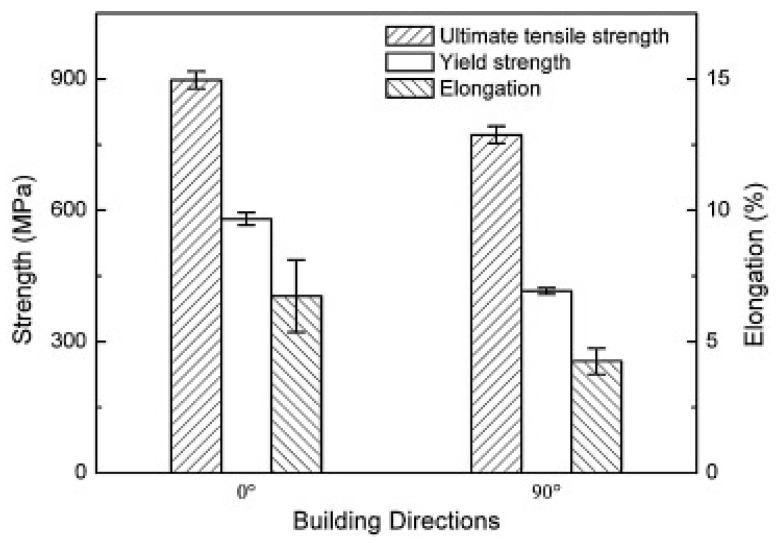
The mechanical properties of DED-fabricated 316L stainless steel alloy are analyzed at both 0° and 90° orientations (sourced from publisher under permission) [49].

The results indicate that specimens in the 0-degree orientation—where the load is applied along the layers—consistently demonstrate superior tensile properties compared to those in the 90-degree orientation, where the load is applied across the layers (see Figure 1). This trend is consistent with findings from other studies, which report that the zero-degree orientation typically achieves higher ultimate tensile strength (UTS). Accordingly, this build direction is often preferred in applications requiring enhanced tensile performance as shown in Figure 2 [49].

While build orientation significantly influences mechanical properties, the deposition pattern is another crucial factor in optimizing part performance and minimizing defects. Common deposition strategies, such as raster, bi-directional, offset, and fractal patterns, impact the final properties of the manufactured part. Selecting an optimal pattern is essential for reducing residual stresses and mitigating thermal distortion. For instance, the offset pattern has been shown to reduce out-of-plane distortion by nearly one-third compared to the bi-directional pattern. Meanwhile, the raster pattern offers versatility, making it suitable for products of varying shapes, thereby enhancing manufacturing flexibility. Additionally, aligning deposition lines at 90 degrees to the longer axis of the substrate can help minimize part deflection, ultimately improving dimensional accuracy [50,51,52,53].

Both infill density and nozzle diameter, typically in the ranges of 15–25% and 0.20–0.40 mm, respectively, play a crucial role in determining the tensile strength of 316L stainless steel fabricated using the DED process. An increase in infill density generally leads to an improvement in tensile strength. Specifically, for a 0.40 mm nozzle diameter, tensile strength showed an enhancement of 20–30%. However, an unexpected trend was observed, where samples with 15% infill density exhibited higher tensile strength compared to those with 20–25% infill. A similar pattern was noted for a 0.2 mm nozzle diameter, with tensile strength consistently improving as infill density increased [54].

Lower laser power combined with high traverse speed reduces energy input at the upper section of the sample, leading to finer microstructures due to accelerated cooling. Conversely, when traverse speed is decreased, and laser power is increased, the cooling rate slows down, promoting the development of coarser microstructures [55,56,57,58]. Research suggests that a higher energy density typically improves the compressive stiffness of 316L stainless steel. However, defining a direct correlation between energy density and porosity in the DED process remains complex. Additionally, as the build height increases, porosity tends to decrease due to a reduction in cooling rates in the upper layers. Among key process variables—such as laser power, scan speed, and feed rate—laser power exerts the greatest influence on the alloy’s mechanical properties [59]. In a separate study, adjusting the scan speed within the range of 960 mm/min to 1200 mm/min had minimal impact on yield strength and elongation. However, variations in laser power were found to significantly influence these mechanical properties [60].

Another experiment revealed that lower laser power (around 400 W), combined with a high scan speed (up to 10 mm/s) and a feed rate of 10 g/min, relative to other DED parameters values, significantly improved the mechanical properties, microhardness, friction behavior, and modulus of 316L stainless steel [61]. A low feed rate ensures complete melting of the material, improving mechanical properties. However, it increases processing time. In contrast, a higher feed rate speeds up production but may lead to defects such as porosity and lack of fusion, reducing tensile strength. Therefore, an optimized feed rate is essential to balance mechanical properties and processing efficiency [62,63,64].

During a DED experiment involving 316L stainless steel, single tracks were deposited onto the base plate at different preheating temperatures—25 °C, 200 °C, 300 °C, 400 °C, and 500 °C. The findings revealed that as the preheating temperature increased, grain size also grew. This grain enlargement contributed to reducing residual stress and enhancing fatigue resistance, ultimately improving the material’s overall mechanical performance [65]. A recent study compared 316L stainless steel samples deposited on both preheated and cold substrates. It was observed that preheated substrates resulted in lower thermal gradients and cooling rates, which further decreased as the layer height increased. In contrast, samples deposited on cold substrates exhibited higher tensile strength and hardness. However, the cold substrate samples showed more non-metallic inclusions within the microstructure, while the slower cooling rate in preheated samples led to reduced formation of δ-ferrite content [66]. Another study found similar trends, showing that different preheating temperatures (room temperature vs. 300 °C) resulted in comparable residual stress and hardness values. However, samples preheated at 300 °C exhibited fewer defects, likely due to improved thermal stability and reduced cooling rates during the DED process [67]. Preheating has been shown to reduce defects in DED processes. Researchers have implemented a preheating technique using a laser-based moving heat source on the substrate, integrated into a Dmg Mori Lt 65 Ded Hybrid Machine, enhancing the quality of the deposition [68].

Studies have shown that increasing the shielding gas flow rate from 5 L/min to 25 L/min, specifically using argon or argon with 3% nitrogen, reduces the oxygen content in the melt pool during the DED process, thereby influencing the formation of oxides and affecting mechanical properties such as strength and ductility [69,70]. Research on Directed energy deposition (DED) of 316-grade stainless steel highlights the crucial role of shielding gas mixtures in influencing the final properties of materials. An argon-based mixture with 3% nitrogen was found to offer superior results, delivering higher tensile strength with significantly less variability. This improvement is linked to a refined microstructure and reduced defect formation. On the other hand, using He-Ar-CO_2_ or Ar-CO_2_ mixtures led to a drop in tensile strength and an increase in porosity. The increased porosity and scatter in mechanical properties for these mixtures are mainly due to the stabilization of δ-ferrite during solidification, which compromises the overall material performance [71]. The discussion highlights key DED process parameters and their influence on the mechanical properties of 316L stainless steel. Table 3 summarizes the optimized parameters based on the above discussion and their corresponding effects.

### 2.2. Selective Laser Melting (SLM)

Several parameters discussed in this section significantly influence material properties. Among these, build orientation is particularly critical in selective laser melting (SLM) fabrication, where a 45-degree orientation demonstrates higher tensile strength, while a 90-degree orientation exhibits greater elongation before failure. Additionally, the fatigue limit at 3 × 10^5^ cycles indicates that the 45-degree orientation has superior fatigue resistance compared to the 90-degree orientation [72]. SLM-fabricated 316L stainless steel parts exhibit distinct mechanical properties based on build orientation. Horizontally built dog-bone specimens show higher ductility, while vertically built ones demonstrate greater strength, emphasizing the critical role of build direction in mechanical performance [73,74]. The referenced study selected a 90-degree build orientation for one of the experiments due to its lower crack density compared to 0-degree and 45-degree orientations [75]. Research showed that hardness is highest at a 90-degree orientation, where reduced layer thickness results in the formation of fine crystalline structures [76].

Meanwhile, electron backscatter diffraction (EBSD) analysis of scanning strategies for 316-grade stainless steel revealed the formation of 〈100〉-oriented grains, with ±45° inclined cells adopting a 〈101〉 orientation. This structural development plays a crucial role in enhancing mechanical properties. Applying scan rotation removed the 〈001〉-oriented grains and increased the frequency of high-angle boundaries. Scanning at 45° and 67° rotations resulted in an increased presence of high-angle grain boundaries. Samples fabricated without scan rotation exhibited a tensile strength of 527 ± 5.4 MPa, a yield strength of 449 ± 2.4 MPa, and a ductility of 58 ± 1.3%. When a 67° scan rotation was applied, tensile strength decreased to 485 ± 4.8 MPa, yield strength dropped to 427 ± 5.4 MPa, and ductility was measured at 50 ± 1.3% [77].

A study found that a strong 〈110〉 texture along the build direction enhances ductility through twinning, while the 90-degree orientation exhibits lower ductility but higher strength [78]. Also, the scanning strategy, along with process parameters like hatching patterns (alternate, cross, one direction), beam passes (single, double, etc.), and scan paths, significantly influence the material properties. Variations in hatching and scanning strategies help in controlling the mechanical properties. The rectangular and hexagonal scanning patterns demonstrated epitaxial grain growth without layer rotation, but the application of rotation disrupted this growth. A 47-degree rotation produced finer grains and enhanced mechanical properties compared to the 90-degree rotation. Furthermore, the rectangular scan pattern resulted in greater hardness than the hexagonal pattern [79]. Reducing hatch spacing enhances overlap between melt pools, which decreases porosity and increases microhardness. Wider hatch spacing, while reducing processing time, can result in poor fusion and increased defects, negatively impacting mechanical properties. Achieving an optimal balance in hatch spacing is vital for ensuring uniform melting and improving part quality in SLM-fabricated 316L stainless steel [80]. In addition to this, high scanning speeds can increase tensile strength by refining the microstructure but may result in reduced density due to incomplete melting and higher porosity. Conversely, lower scanning speeds allow for thorough melting, resulting in deeper melt pools, improved fusion, and reduced porosity. An optimal scanning speed is essential to balance tensile strength and ductility, preventing defects like microcracks and ensuring stable microstructural formation [81,82].

Studies indicate that a 60 µm layer thickness reduces hardness variation, though the hardness value itself may be slightly lower. In contrast, 30 µm, 80 µm, and 100 µm thicknesses show better hardness but lead to higher surface roughness, increasing the risk of defects [83]. Layer thicknesses in the range of 30 µm to 50 µm lead to variations in the internal microstructure, significantly altering the properties of 316L stainless steel [84]. Increasing the layer thickness reduces both relative density and hardness due to limited laser penetration. This results in less effective melting, weaker interlayer bonding, and increased defects, ultimately compromising the mechanical properties [80].

Optimizing other parameters such as energy density is crucial as it can enhance mechanical properties by minimizing porosity. Increasing energy density improves densification and microhardness by reducing defects and strengthening layer bonding. Hardness increases linearly with energy density from 50 to 125 J/mm^3^, but exceeding this range can cause coarsening of the cellular microstructure, reducing hardness and diminishing resistance to local deformation [85,86,87]. While higher laser power generally enhances fusion, boosting tensile strength and density, excessive energy input may cause material vaporization, leading to issues such as keyhole porosity (laser-induced void) and grain coarsening, which deteriorate hardness and mechanical strength. Further, excessive power can also lead to spatter, compromising surface quality. High-power lasers achieve similar densities to low-power ones but tend to form wider and shallower melt pools, resulting in a coarser microstructure and increased cracking due to thermal gradients. In contrast, lower power produces deeper and narrower melt pools, promoting finer grain structures, which contribute to better hardness and enhanced mechanical properties [72,75,80,82,88]. Moreover, smaller laser spot sizes focus energy more effectively, resulting in a finer-grain microstructure with higher resolution [82].

Preheating the build plate to 150 °C helped form an equiaxed microstructure while reducing porosity due to lower cooling rates. This modification increased ductility by 14% and produced a more uniform structure, potentially improving fatigue performance [89]. Preheat-treated specimens exhibited a 62% increase in fatigue life and a 12.35% reduction in cyclic softening compared to the without-preheat-treatment (WHT) specimens. The preheated specimens also showed fewer pores and improved material fusion, contributing to their overall superior fatigue resistance [90]. Studies have indicated that preheating the build platform in SLM reduces residual stresses, enhances strength, and improves hardness [91]. Studies indicate that powder bed preheating in SLM reduces thermal stresses in tool steel materials and results in a more uniform microstructure. Preheated samples exhibit superior mechanical properties, including higher hardness and tensile strength, compared to non-preheated samples [92].

Similarly, at a low shielding gas flow of 500 L/min, inadequate removal of by-products causes defects such as porosity and poor interlayer fusion. Increasing the flow rate to 550–600 L/min significantly enhances the removal of contaminants, reduces powder bed pollution, and improves bonding, leading to better tensile strength and uniform material properties [93]. Flow rates below 500 L/min risk oxidation and powder erosion, leading to defects such as porosity and poor layer bonding [94]. Studies indicate that argon gas effectively minimizes oxidation and enhances toughness. In contrast, nitrogen can form nitrides, increasing hardness but potentially reducing ductility. The use of Helium in SLM reduces the interaction between the laser energy and the powder bed. This leads to obstruction of the laser beam by a large plasma plume, limiting effective energy transfer. As a result, the mechanical properties of the 316L stainless steel alloy are compromised, with increased porosity and lower part density [95].

Balancing these process parameters is essential for minimizing defects and optimizing mechanical properties. Optimizing process parameters such as laser power, scanning speed, and energy density enhances densification, microhardness, and mechanical performance, ensuring superior reliability and durability in SLM-fabricated 316L stainless steel components [96]. Table 4 summarizes the optimized SLM process parameters discussed above, along with their recommended ranges and corresponding effects on mechanical properties, to guide parameter selection for achieving desired material performance.

### 2.3. Electron Beam Melting (EBM)

**Figure 3:**
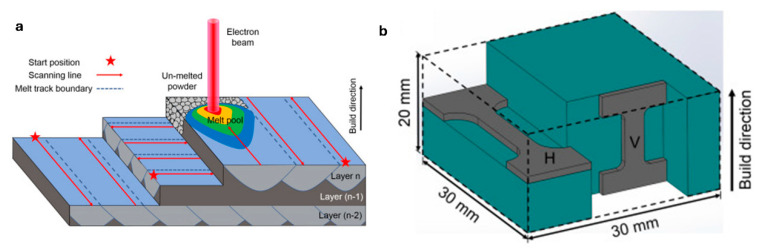
Electron beam melting (EBM) scanning strategy (**a**) and build orientations for 316L stainless steel: horizontal and vertical build directions (**b**) (sourced from publisher under permission) [97].

To optimize the mechanical properties of the fabricated components, electron beam melting (EBM) was employed using a distinct three-layer scanning pattern. The scanning pattern is depicted in Figure 3, where red arrows indicate the specific scanning strategy implemented. To investigate the influence of build direction on material properties, samples were fabricated in two different orientations, as illustrated.

1. Horizontal-build-direction samples are built with layers oriented parallel to the build plate.
2. Vertical-build-direction samples are built with layers oriented perpendicular to the build plate.

This approach allowed for a comparative analysis of the tensile properties, particularly tensile strength, of the 316L stainless steel samples. The tensile tests were performed on both horizontally and vertically fabricated samples to assess the anisotropy in mechanical properties resulting from the layer-wise build process and scanning strategy [97].

**Figure 4:**
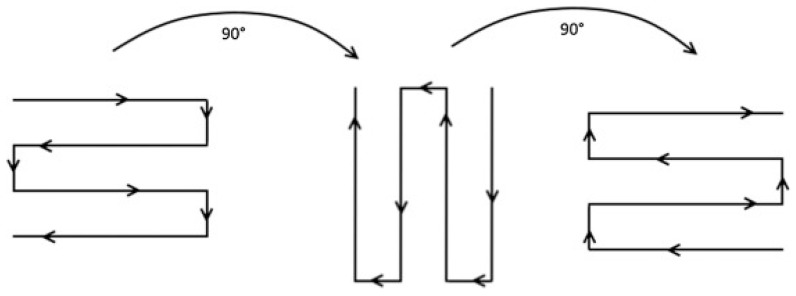
Illustration of the modified scan pattern, rotated by 90 degrees, and the corresponding preheating conditions (800–820 °C) used in the EBM process for 316L stainless steel. These adjustments contributed to enhanced material properties and better consolidation (sourced from publisher under permission) [100].

In the tensile testing of EBM-manufactured SS316L samples, the results showed greater tensile strength compared to conventional cast and wrought 316L stainless steel, but the ductility was notably lower. The EBM-fabricated SS316L samples also exhibited strong anisotropic tensile properties. It was observed that the samples had better tensile strength in the vertical orientation as compared to the horizontal orientation. During the experiment, the build plate was preheated to temperatures up to 850 °C, where microstructural and fractographic analyses revealed a significant amount of sigma (σ) phase precipitating at the grain boundaries, especially at higher build temperatures. The presence of this phase was found to be more pronounced under conditions of lower scanning factor (SF) and larger focus offset (FO), which contributed to a reduction in tensile properties, particularly for samples built in the horizontal direction. In the build direction, near the top of the EBM-fabricated sample, intragranular cellular sub grains were observed. Similar microstructural features have also been reported in other studies using both SLM and EBM processes, indicating consistent results across different additive manufacturing methods [39,97,98,99,100,101,102]. To further investigate the effect of scanning strategy and preheating, the scan pattern was rotated by 90 degrees (Figure 4), and the build plate was preheated to a temperature range of 800 to 820 °C. This modification resulted in significantly improved mechanical properties, demonstrating superior performance compared to powder metallurgy methods such as hot isostatic pressing (HIP) [100].

Studies also verified and showed that preheating the build plate at higher temperatures reduces defects such as residual stresses for metallic materials [91,103,104]. Similarly, narrower hatch spacing improves part density by promoting effective overlap, while wider spacing increases porosity due to unmelted regions and high scanning speeds often result in insufficient melting, reducing the density of the part. In contrast, lower scanning speeds deliver more energy per unit area, enhancing material consolidation, but can lead to defects if combined with high laser power. Additionally, while volumetric energy density (VED) is a useful metric for assessing porosity and unfused powder, studies indicate that it does not reliably predict final part density, hardness, or microstructural changes in 316L stainless steel [100]. Instead, optimizing scanning speed, beam spot size, and hatch spacing plays a more direct role in controlling these properties [105].

Moreover, typical ranges for EBM include beam speeds around 4350 mm/s, beam currents between 1 and 50 mA, and layer thicknesses around 70 μm. These parameters are critical for achieving dense components without cracks or deformations. Fine-tuning these settings is necessary to optimize tensile strength and mechanical properties [106,107]. An investigation into optimal parameters for 316L stainless steel highlighted the importance of controlling beam spot size, scanning speed, and hatch spacing. The study identified optimal process parameters that resulted in a maximum tensile strength of 1491.51 MPa at an energy density of 64.28 J/mm^3^. These parameters included a line offset of 0.1 mm, a layer thickness of 50 μm, a scanning speed of 2800 mm/s, and a beam current of 15 mA, all of which contributed to enhancing the material’s mechanical performance. These parameters provided the best balance between density, microstructure, and mechanical strength [108]. The beam spot size was found to significantly affect the melt pool width, influencing microstructural consistency and melt pool overlap. A smaller beam spot size promotes better control of the melt pool, leading to consistent microstructural evolution and enhanced part quality [105]. Energy input in EBM is governed by multiple factors, including beam voltage, scanning speed, beam current, and track offset distance. Studies have shown that adjusting these parameters within an intermediate range results in the best mechanical properties. Fine-tuning these settings is essential to achieving maximum tensile strength, as demonstrated in maraging steel under optimized process conditions [109].

In addition to process parameters, for electron beam melting (EBM), operating in a vacuum environment is essential to minimize electron beam attenuation and ensure efficient energy deposition. However, this vacuum can lead to the evaporation of volatile elements in the alloy due to their high vapor pressures. Increasing the helium (He) pressure within the vacuum chamber can enhance the thermodynamic stability of these elements, reducing their tendency to evaporate. Monte Carlo simulations and thermal experiments indicate that increasing He pressure from 10^−3^ mbar to 10^−2^ mbar results in less than a 1.5% decrease in energy deposition efficiency. These findings suggest that controlled gas pressures can be strategically employed to process alloys with volatile elements while maintaining EBM process efficiency [110].

Table 5 presents the optimized process parameters (based on the above discussion) for electron beam melting (EBM) of 316L stainless steel, including recommended values for each parameter and their corresponding effects on mechanical properties. The provided ranges help researchers select optimal conditions to enhance material strength, reduce defects, and improve overall part quality.

### 2.4. Process Parameters and Thermodynamic Phase Evolution

Although the influence of thermal gradients and cooling rates has been extensively discussed in Section 4, a deeper thermodynamic analysis of phase evolution during additive manufacturing (AM) of 316/316L stainless steel is crucial before getting into further details relevant to microstructural analysis. The rapid thermal cycling and localized heat input inherent to AM significantly influence the formation and stability of various phases, including δ-ferrite, sigma (σ) phase, and carbides, which can alter mechanical, corrosion, and long-term performance properties.

- δ-Ferrite Formation

During solidification, particularly under high cooling rates or elevated energy input, 316/316L stainless steel can deviate from the equilibrium austenitic solidification pathway and form δ-ferrite as a residual high-temperature phase. This is especially pronounced in techniques like DED and EBM where slower cooling allows for ferritic solidification modes. The formation of δ-ferrite is influenced by Cr_eq_/Ni_eq_ ratios (see Equations (1) and (2) [111]; if ratio ≥ 1.5, it is more likely to form primarily ferrite; if ratio is 1.3–1.5, then austenite + δ-ferrite will be present; and if ratio ≤ 1.3, then fully austenite will be formed) [112] and local composition shifts due to segregation during melting. While δ-ferrite can enhance hot cracking resistance, its presence beyond 5–10% may reduce toughness and corrosion resistance [66,71].

Cr equivalent = %Cr + 1.4 × %Mo + 1.5 × %Si + 0.5 × %Nb + 2%Ti (1)

Ni equivalent = %Ni + 30 × %(C + N) + 0.5 × %M (2)

Studies have shown that the use of argon–nitrogen shielding gas in DED leads to δ-ferrite stabilization due to altered solidification dynamics, while preheated builds promote more uniform austenitic structures by reducing thermal gradients and facilitating diffusion-driven phase transformation [66,71].

- Sigma (σ) Phase Precipitation

The sigma phase is an intermetallic compound rich in chromium and molybdenum that forms between 600 °C and 900 °C temperatures that are relevant during slow cooling or prolonged exposure in EBM and post-processing heat treatments. In AM, σ-phase formation is more likely in regions experiencing intermediate reheating, such as overlapping scan zones or during high-temperature preheating in EBM (>800 °C). For example, cited study [97] reports σ-phase precipitation along grain boundaries in EBM-built 316L samples, correlating with reduced ductility in horizontally fabricated parts.

The sigma phase is known to embrittle the material, reduce toughness, and degrade pitting corrosion resistance by depleting Cr and Mo from the matrix. Its suppression typically requires rapid cooling or solution heat treatment above 1050 °C, followed by water quenching [100,103].

- Carbide Precipitation

Carbide formation, particularly chromium-rich M_23_C_6_ at grain boundaries, is another key transformation observed in 316/316L stainless steel when exposed to sensitization temperatures (500–800 °C). While 316L’s low carbon content reduces this risk, repeated thermal cycling in AM or inadequate post-processing may promote carbide precipitation. This can lead to intergranular corrosion susceptibility.

High scan speeds and lower energy input in SLM typically avoid prolonged exposure to sensitization ranges, minimizing carbide formation. However, in DED and EBM, the slower solidification or thermal accumulation in thick parts may promote carbide segregation, especially without controlled cooling [100].

- Influence of Important Process Parameters on Phase Stability
  (1) High energy density promotes remelting and homogenization, reducing segregation that fosters δ-ferrite or σ-phase formation.
  (2) Low scan speeds and high preheating temperatures (especially in EBM) may increase the likelihood of σ-phase and coarse carbide precipitation unless countered by optimized beam parameters [97,100,106].
  (3) Post-processing treatments such as solution annealing (>1050 °C) followed by rapid quenching can dissolve the σ-phase and carbides, restoring full austenitic structure and improving corrosion resistance and ductility [103,104].

However, phase evolution in AM-fabricated 316/316L stainless steel is essential for predicting alloy performance under thermal or corrosive service. δ-ferrite, σ-phase, and carbides can be beneficial or detrimental depending on their quantity, morphology, and distribution each of which is process-dependent. Future studies should integrate thermodynamic simulations with in situ thermal monitoring to predict and control phase transformations more precisely.

### 2.5. Process Optimization Strategies for Cyclic Thermal and Corrosive Environments

When additive manufacturing (AM) components are intended for cyclic thermal or corrosive service environments such as those in the aerospace, marine, or chemical industries traditional process optimization must be extended to address long-term degradation mechanisms, particularly thermal fatigue, corrosion, and environmental stress cracking. Each AM technique, directed energy deposition (DED), selective laser melting (SLM), and electron beam melting (EBM), requires tailored strategies for such demanding conditions.

In DED, large melt pools and slower cooling rates often lead to coarse grain structures and high residual stresses, increasing vulnerability to crack initiation under thermal cycling [113]. To mitigate this, substrate preheating, use of optimized deposition patterns, and incorporation of interlayer dwell times are critical for reducing thermal gradients and associated stress buildup. Furthermore, post-build heat treatment refines grain boundaries and improves dimensional stability under repeated heating and cooling [66,114,115,116]. In corrosive environments, DED parts exhibit higher surface roughness and elevated porosity, making them more susceptible to pitting and stress corrosion cracking. Optimizing the shielding gas composition such as using argon with nitrogen additions can reduce oxide formation and enhance mechanical and electrochemical performance. Post-processing methods like electropolishing or laser remelting further minimize surface irregularities that act as corrosion nucleation sites [114,117,118,119]. In SLM, the optimization must address high thermal gradients that generate significant residual stresses and columnar grains. These issues are especially problematic under thermal cycling, where microcrack formation can propagate through weak interlayer bonds. Preheating the build platform, refining scan strategies, and applying heat treatment can improve ductility and reduce fatigue failure risks [99,114,118,120]. For corrosive resistance, dense microstructures, low porosity, and smooth surfaces are crucial. This can be achieved through optimized energy density, reduced hatch spacing, and argon-based shielding gas flow, followed by surface passivation or polishing [87,94,114,121,122]. On the other side, EBM, with its high preheating temperatures and vacuum operation, inherently minimizes residual stress accumulation and oxidation. However, coarse surface morphology, layer thickness, and limited resolution can still contribute to thermal fatigue under cyclic loads. Rotating scan strategies and optimized beam parameters are essential to ensure fine microstructure and uniform energy distribution. For corrosion-critical applications, EBM’s vacuum environment reduces contamination, but post-build finishing remains necessary to smooth surface roughness and eliminate subsurface pores [16,97,123].

However, optimizing AM processes for cyclic or corrosive environments requires a multi-objective approach, targeting not only mechanical strength but also microstructural stability, residual stress control, porosity reduction, and surface refinement. By integrating thermal management, strategic deposition planning, gas control, and post-processing, each AM technique can be adapted to meet the stringent durability requirements of real-world operating conditions.

### 2.6. AI/ML in Optimizing Parameters and Predictions of Defects and Properties

Machine learning (ML) and artificial intelligence (AI) have become central to addressing key challenges in additive manufacturing (AM), especially for 316/316L stainless steel. These include optimizing process parameters, predicting mechanical properties, and enabling real-time process monitoring. Recent studies [124,125,126,127,128,129] emphasize the effectiveness of supervised learning techniques, including artificial neural networks (ANNs), random forest, Gaussian process regression (GPR), and SVM, for process parameter optimization in laser powder bed fusion (LPBF) and directed energy deposition (DED) processes. These models correlate critical parameters—like laser power, scan speed, layer thickness, and hatch spacing—with part quality metrics such as density and surface roughness. For example, ref. [130] developed a universal predictor-based ML model using volumetric energy density to enhance model transferability across different machines and materials, achieving up to 80% prediction accuracy for relative density and surface finish even with small datasets.

AI-driven systems also enhance real-time monitoring by integrating in situ sensor data. The cited study [131] demonstrated a computer vision algorithm for LPBF machines that can classify powder bed anomalies during the recoating process, potentially enabling closed-loop control systems to correct deviations as they arise. Researchers also reviewed how digital twins, virtual replicas of the build process, can use ML algorithms to monitor and adjust builds in real time, improving part quality and minimizing defects [130].

ML also facilitates predictive modeling of mechanical outcomes such as yield strength, tensile strength, and hardness. Researchers developed a vertically integrated modeling framework for DED, which combines powder flow, molten pool dynamics, and residual stress analysis to predict microstructure and mechanical behavior with high fidelity. These models significantly reduce reliance on trial-and-error experimentation by enabling virtual testing environments and process optimization based on predefined property targets [32].

Researchers noted that AI techniques are essential for scaling AM across industries, allowing data-driven decisions for material selection, process settings, and post-processing strategies. Integration of AI with sensor-rich environments supports Industry 4.0 objectives, reducing waste, improving quality, and enabling sustainable manufacturing practices. AI and ML are no longer supplementary but core technologies in AM. Their ability to optimize parameters, predict mechanical properties, and enable real-time feedback mechanisms is reshaping how high-performance materials like 316L stainless steel are manufactured [132,133].

### 2.7. Critical Evaluation and Comparison of Mechanical Properties

#### Qualitative and Quantitative Framework

To determine the most effective additive manufacturing process for 316/316L stainless steel, a comparative critique of directed energy deposition (DED), selective laser melting (SLM), and electron beam melting (EBM) has been conducted. Each process presents unique advantages and limitations, influencing its suitability for specific industrial applications—for example, DED allows for large-scale component repair but suffers from poor surface finish; SLM offers high precision and excellent mechanical properties but is limited by build size and high residual stresses; whereas EBM minimizes residual stress through high preheating but is constrained by lower resolution and material compatibility. A critical evaluation of mechanical strength, microstructural integrity, porosity, and residual stress formation is essential for making an informed selection.

- Directed Energy Deposition (DED)

DED is often chosen for large-scale component fabrication due to its high deposition rates. It allows for rapid prototyping and cost-effective material usage, particularly in applications where modifying or repairing existing parts is necessary. The flexibility in feedstock types, including wire and powder, further enhances its adaptability. However, the process lacks precision compared to other additive manufacturing techniques, as it produces coarser surface finishes, requiring extensive post-processing. This limitation makes DED less suitable for applications demanding intricate geometries. A major drawback of DED is the formation of residual stresses due to rapid cooling. These stresses contribute to structural distortion, necessitating stress-relief treatments. Additionally, the presence of porosity and fusion defects undermines mechanical integrity, making it unsuitable for applications requiring high fatigue resistance. Preheating the build plate (200–500 °C) has been shown to reduce residual stress formation and porosity, but this does not completely eliminate anisotropy in mechanical properties. The cited studies showed components printed in vertical orientations tend to exhibit higher tensile and yield strengths compared to those fabricated in horizontal orientations. However, the uneven cooling rates characteristic of DED makes it difficult to achieve uniform material properties across the entire part. While DED is advantageous for large components and repair applications, its lack of precision and high residual stress levels makes it a less favorable choice for industries requiring fine feature resolution and high structural reliability [48,61,63,65].

- Selective Laser Melting (SLM)

SLM is widely regarded as the most precise additive manufacturing technique for stainless steel, allowing for the production of intricate geometries with high part density. The fine microstructure achieved in SLM results in enhanced mechanical strength, making it an attractive choice for aerospace, biomedical, and high-performance engineering applications. However, the process is highly sensitive to parameters selection. Factors such as energy density, scanning speed, and hatch spacing must be precisely regulated to prevent defects such as porosity and keyholing. Failure to optimize these parameters can lead to a significant decrease in mechanical performance. High residual stresses are another concern, as they can lead to part warping and cracking, especially for larger builds. Unlike DED, SLM is limited by build size constraints, restricting its use in applications requiring large-scale manufacturing. Moreover, operational costs are significantly higher due to the need for strict environmental control and post-processing to achieve the desired surface quality. Thermo-mechanical behavior in SLM is primarily governed by energy density, which directly influences part density and hardness. Preheating the build plate to approximately 150 °C mitigates residual stresses but does not fully resolve them. The choice of layer thickness is another critical factor—while thinner layers enhance part density, they increase surface roughness, requiring additional finishing processes. SLM is the preferred choice for applications requiring high precision, strength, and fine microstructures, but it is not ideal for large-scale manufacturing due to size limitations, and the high costs associated with maintaining strict process parameters [72,85,87,91].

- Electron Beam Melting (EBM)

EBM offers a unique advantage over DED and SLM by operating in a vacuum environment, preventing oxidation and enhancing material purity. This makes it an excellent choice for applications requiring high-performance components with minimal contamination. One of the most significant benefits of EBM is its ability to preheat materials to approximately 850 °C, effectively reducing residual stresses and minimizing part distortion. This aspect makes EBM particularly suited for manufacturing components that require high structural stability, such as those in the aerospace and medical industries. However, EBM has notable drawbacks. The process has a lower resolution compared to SLM due to larger beam spot sizes and thicker layers, making it unsuitable for applications requiring fine feature details. Additionally, material compatibility is limited because of the vacuum environment, restricting the range of alloys that can be effectively processed. EBM also suffers from high setup and operational costs, making it a less economical choice for industries that do not specifically require its unique advantages. Thermo-mechanical effects in EBM are heavily influenced by preheating, which significantly reduces residual stresses and improves thermal stability. Build orientation plays a crucial role in anisotropy, with vertical builds generally exhibiting higher tensile strength. However, excessive energy input can lead to sigma phase precipitation at grain boundaries, reducing ductility and potentially compromising mechanical performance. While EBM is the most effective process for residual stress reduction and maintaining material purity, its lower resolution, limited material compatibility, and high costs make it a specialized rather than a widely applicable additive manufacturing technique [91,97,106].

- Which Process is the Best for 316/316L SS?

The choice between DED, SLM, and EBM depends on the specific requirements of the application. For large-scale components where deposition speed is a priority, DED is the most suitable choice, despite its limitations in precision and surface finish. For applications requiring high precision, fine microstructures, and superior mechanical strength, SLM is the optimal choice, provided that the limitations in build size and residual stresses are managed. For industries prioritizing stress reduction and material purity, EBM is the best option, though its high costs and lower resolution limit its use to specialized applications. However, EBM effectively reduces residual stresses but lacks the resolution and material flexibility of SLM and DED. Each of these additive manufacturing processes has distinct strengths and weaknesses, making it crucial to align process selection with specific engineering requirements. For researchers and engineers selecting an additive manufacturing process for 316/316L stainless steel, SLM remains the best overall choice for achieving optimal mechanical properties and part quality, provided that the challenges of residual stress and cost are addressed through effective process control and post-processing techniques [48,61,63,65,72,85,87,91,97,106].

- Mechanical Properties Comparison (AM vs. Conventional)

Table 6 presents a comprehensive comparison of process parameters and their impact on the mechanical properties of 316/316L stainless steel across different manufacturing techniques. These techniques include directed energy deposition (DED), selective laser melting (SLM), electron beam melting (EBM), and conventional processing methods such as wrought and cast production. The comparison focuses on critical parameters such as build orientation, energy input, scanning speed, and preheating conditions, evaluating their effects on ultimate tensile strength, yield strength, elongation, and hardness. By incorporating the technique (orientation) column, the table provides a clearer understanding of how specific processing conditions influence material behavior.

**Table 6:** Mechanical properties of 316/316L stainless steel based on additive manufacturing process parameters—comparison of DED, SLM, EBM, and conventional methods (wrought, cast).

Technique and Build Orientation	UTS (MPa)	Yield Strength (MPa)	Elongation (%)	Hardness (HV)	Process Parameters	Refs.
DED (X)	776	576	33%	289	Laser power: 400 W, V: 15 (mm/s)	[134]
DED (Y)	703	479	46%	272	Laser power: 400 W, V: 15 (mm/s)	[134]
DED (90°) Overlap in X: 50%, Z: 25%	469 ± 6	649 ± 2	23 ± 3	-	Laser power: 900 W, V: 15 (mm/s)	[135]
DED (67°) Overlap in X: 50%, Z: 25%	469 ± 10	624 ± 10	17 ± 3	-	Power (P): 900 W, speed (υ): 15 mm/s	[135]
SLM (X)	666–738	653–718	20–36%	235	Laser power: 200 W; V: 1000 (mm/s), spot size: 80–300 μm, layer thickness; 30–100 μm	[136]
SLM (Y)	653–680	541–668	30–33%	235	-	[136]
SLM (Z)	555–608	508–577	40–47%	235	-	[136]
EBM (X)	571.8 ± 19.3	334.2 ± 15.5	29.3 ± 5.2	-	Layer thickness: 50 μm, plate temperature: 850 °C	[97]
EBM (X) Another sample	436.5 ± 23.2	342.9 ± 22.8	9.6 ± 2.3	-	-	[97]
EBM (Y)	580.2 ± 6.8	315.7 ± 10.0	35.2 ± 2.3	-	-	[97]
EBM (Y) Another sample	651.7 ± 8.5	395.8 ± 9.0	30.6 ± 3.0	-	-	[97]
Wrought (316)	586	234	50	160–200	-	[55]
Wrought (316L)	480	170	40	140–190	-	[55]
Cast (316)	485–585	240–290	30–35	150–180	-	[137,138,139]
Cast (316L)	450–550	200–250	30–35	140–170	-	[137,138,139]

- Process Parameters with the Highest Reported Variability in Mechanical Properties

Across the reviewed studies as discussed above, laser power emerged as the process parameter associated with the highest variability in mechanical property outcomes for 316/316L stainless steel components manufactured using DED, SLM, and EBM. In DED, changes in laser power—from as low as 400 W to 900 W—resulted in substantial variation in tensile strength (469–776 MPa) and elongation (17–46%), indicating a strong influence on melt pool behavior, grain morphology, and porosity. Similarly, in SLM, even moderate adjustments in laser power (e.g., 200–220 W) significantly affected yield strength (508–718 Mpa) and elongation (20–47%), particularly when interacting with build orientation and scanning strategies. In EBM, beam current (functionally equivalent to laser power) directly influenced density, grain coarsening, and defect formation, thus impacting tensile and ductile responses. While other parameters like scan speed and hatch spacing contributed to mechanical differences, the reviewed data consistently showed laser power as the dominant factor contributing to variability in mechanical performance across additive manufacturing processes.

- Decision Matrix and Performance Index to synthesize mechanical, economic, and processing feasibility of DED, SLM, and EBM

To reinforce the comparative findings presented in this review, a decision-support tool was developed by introducing a multi-criteria decision matrix and corresponding performance index (PI). This framework synthesizes mechanical, economic, and process-related factors, offering a consolidated view of how directed energy deposition (DED), selective laser melting (SLM), and electron beam melting (EBM) perform when applied to 316/316L stainless steel fabrication.

Each AM process (DED, SLM, and EBM) was scored across eight evaluation criteria using a 1–5 scale, where 5 represents the best observed performance. These scores were drawn directly from Table 1, Table 2, Table 3, Table 4, Table 5, Table 6, Table 7 and Table 8 and the discussion sections of this review, which compare process parameters, microstructural outcomes, post-processing needs, and mechanical behavior. The matrix was built using a weighted sum model, a common method in engineering design and materials selection decision-making, as Equation (3) explains [140,141].

(3) $${PI}_{j}=\sum_{i=1}^{n}\omega_{i}.S_{i.j}$$

where

*PI_j_* is the performance index of AM process *j*.

*ω* is the weight of criterion *i*.

*S_ij_* is the score of process *j* for criterion *i*.

To reflect scenarios where mechanical strength and surface quality are especially critical, such as in aerospace, biomedical, and precision tooling applications, the evaluation criteria were reweighted accordingly.

The table below integrates the evaluation criteria, their assigned weights, rationales, and the comparative scores of directed energy deposition (DED), selective laser melting (SLM), and electron beam melting (EBM) across each criterion. The scores are derived from detailed analysis in this review, and the weights reflect their importance in high-performance applications. The performance index (PI) is calculated using a weighted sum model, providing a single score for each method.

**Table 7:** Integrated decision matrix with weighted criteria, rationales, and performance index for additive manufacturing methods applied to 316/316L stainless steel.

Criterion	Weight	Rationale	DED	SLM	EBM
Mechanical Strength	0.25	High-priority for structural reliability	3	5	4
Surface Finish	0.15	Crucial for fatigue, sealing, and part interfaces	2	5	3
Porosity/Defect Control	0.10	Key for part integrity and fatigue resistance	3	4	5
Residual Stress Reduction	0.05	Supports dimensional stability, but less critical when post-processed	3	2	5
Build Speed	0.10	Relevant in high-throughput production	4	2	4
Dimensional Accuracy	0.10	Required in assembly-critical or complex geometry applications	2	5	3
Post-Processing Requirements	0.10	Affects lead time, cost, and workflow complexity	3	2	5
Economic and Operational Cost	0.15	Important for adoption in manufacturing environments	4	2	3
Performance Index (PI)	-	-	3.00	3.70	3.85

Despite the marginally higher overall PI for EBM, this outcome primarily reflects its superior performance in stress mitigation and defect reduction. However, in contexts where mechanical performance and surface precision are prioritized, SLM achieves nearly equivalent performance (PI = 3.70) and remains the most favorable technique due to its exceptional strength-to-weight ratio, fine resolution, and refined microstructure. This is consistent with the earlier qualitative conclusion identifying SLM as the optimal method for applications demanding tight tolerances and mechanical integrity. DED, with a lower PI of 3.00, offers value in scenarios prioritizing scalability, repair, and cost-efficiency, though with trade-offs in precision and defect sensitivity.

## 3. Defects

### 3.1. Porosity

AISI 316L stainless steel was produced using the directed energy deposition (DED) method to analyze porosity on an S235JR mild steel substrate. The study focused on four specific regions: the top and bottom zones in both the YZ and XZ planes. Porosity was categorized into two ranges: (i) 0.0–20.0 μm and (ii) 21.0–40.0 μm. While all examined areas demonstrated a high material density exceeding 99.80%, hardness variations were observed due to differences in grain size, with lower hardness near the fusion line and higher hardness within the track body. The primary causes of defects were attributed to air entrapment, moisture evaporation, and incomplete melting [142].

Compared to traditional manufacturing techniques, selective laser melting (SLM) achieves higher densification (~99.97%) [143] and improved mechanical performance in 316L stainless steel. An optimized SLM sample processed with specific parameters (P = 170 W, h = 0.08 mm, v = 1000 mm/s) demonstrated the greatest yield strength (421 MPa), hardness (245 HV), and elongation (42%). However, certain defects, such as weak bonding, were linked to low energy density, which limited the penetration of molten metal into previously solidified layers. These irregular defects were primarily caused by insufficient energy input, while spherical gas porosities observed at higher energy densities likely resulted from gas entrapment during atomization or within the processing environment. Additionally, the rapid cooling rates in SLM impeded gas bubble escape from the molten pool, causing their entrapment during solidification [143,144]. These process-induced defects underscore the critical importance of optimizing parameters to mitigate challenges [145,146], while laser power and scanning speed variations in SLM significantly affect the morphology of solidified melt pools, thereby influencing porosity formation in 316L stainless steel. For instance, maintaining consistent energy density while reducing laser power and scanning speed effectively decreased porosity levels. Optimized SLM processes can yield periodic, overlapping melt pools, resulting in samples with minimal porosity. While porosity had a limited impact on strength, ductility demonstrated greater sensitivity. Samples processed at 135 W and 750 mm/s exhibited low porosity and an approximate 10% improvement in ductility, highlighting the necessity for precise parameter control to enhance the mechanical performance of SLM 316L stainless steel [147].

**Figure 5:**
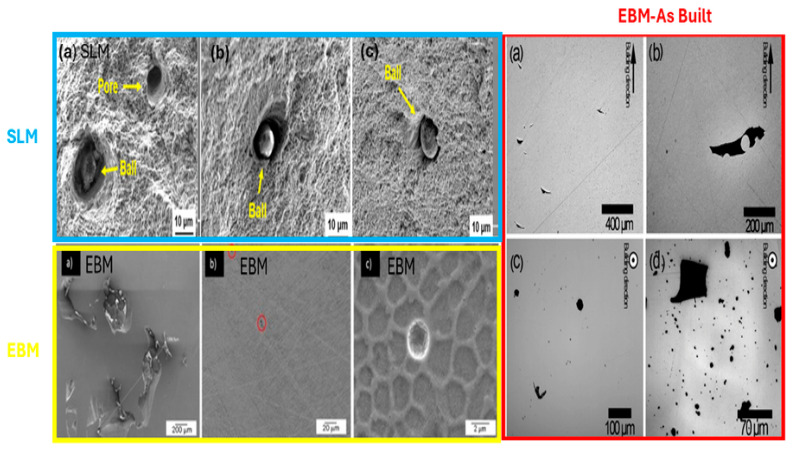
{Selective laser melting (SLM, highlighted blue area) analysis indicates the presence of various defect types influenced by processing conditions. At a laser power of 120 W and a scan speed of 100 mm/s, all samples exhibited pores and balling defects: (**a**) SLM—as built, (**b**) HT-SLM, (**c**) HIP-SLM [143]}. {Electron beam melting (EBM, yellow highlighted area) with a 200 μm layer thickness presents various defects, as observed in SEM images. These include (**a**–**c**) unmelted and partially melted powder particles, cracks, voids, macro-level porosity, and encapsulated gas bubbles. Such defects emphasize the challenges associated with this specific parameter setting [100]}. {Electron beam melting (EBM) of SS316L: The red-highlighted regions illustrate structural characteristics of the as-built component along the designated build direction. (**a**,**b**) The side view exposes the internal structure, along with a noticeable large defect. (**c**,**d**) The cross-sectional view reveals internal flaws, including porosity clusters near the sample edges. All examined areas exhibit voids, porosity, and structural inconsistencies [39]}. (Images reproduced with permission from publisher).

The electron beam melting (EBM) technique used to fabricate 316L stainless steel with thicker layers (200 μm) resulted in increased defects and slightly reduced density and hardness compared to thinner layers (100 μm). This emphasizes the need for parameter optimization for thicker layers to improve material quality. Investigating the effect of layer thickness on fatigue performance is particularly critical for applications involving significant temperature variations and cyclic thermal stresses [96]. Furthermore, an EBM-fabricated SS316L component revealed micron and submicron pores alongside larger crescent-shaped pores up to 200 μm, indicating insufficient energy input and challenges with melt pool overlap. The edges of the sample exhibited higher porosity and lower density compared to the inner regions, with a calculated cross-sectional density of 99.8%. These findings highlight the necessity of optimizing process parameters to improve edge density and overall material quality [39]. The porosity defects associated with various additive manufacturing processes concerning process parameters are summarized in Figure 5.

### 3.2. Surface Roughness

Researchers studied how process parameters affect surface roughness in single-track clads made by DED, comparing the blown powder and wire-fed methods. In the blown powder process, increasing the feed rate led to a noticeable rise in surface roughness, ranging from 8.94 to 38.77 µm for stainless steel. This variation was closely linked to energy input and material deposition rates, with optimal roughness achieved at high energy density and lower material feed rates, ensuring better integration of powder particles into the clad. In contrast, the wire-fed method produced smoother surfaces, with roughness values between 1.00 and 8.33 μm. However, surface quality remained highly sensitive to process conditions, particularly at lower current levels, where unstable material transfer and melt pool dynamics led to increased roughness. The study identified that maintaining a low material feed rate (<0.35 g/m) and energy input below 40 J/mm^3^ effectively minimized defects such as humping (wave-like bulging) and balling (spherical droplet ball formation). These findings highlight the importance of optimizing process parameters to improve surface quality in metal DED techniques [148]. The study further explores how laser power and scanning speed affect surface roughness, clad morphology, microstructure, texture, and hardness of 316L stainless steel in the directed energy deposition (DED) process. While surface roughness exhibited minimal sensitivity to scanning speed, it decreased significantly with higher laser power, highlighting the critical role of energy input in achieving smooth surfaces. Moreover, scanning speed was found to affect grain orientations, while hardness remained largely uniform across conditions, except at high laser power, which resulted in finer microstructures and a blend of austenite and martensite phases [149]. Additionally, the application of DED coating followed by laser scanning treatment demonstrated substantial enhancements in surface smoothness. Roughness values improved from the initial 10.20 μm and 9.80 μm (R_a,x_ and R_a,y_, respectively) of the L-PBF sample to 8.30 μm and 5.60 μm after DED coating. Laser scanning further refined these surfaces, achieving minimum roughness values of 2.10 μm and 4.00 μm, illustrating the effectiveness of combined post-processing treatments in improving surface quality [150]. Further analysis of DED-fabricated 316L SS alloys highlighted the relationship between surface roughness and balling phenomena, as previously discussed in Figure 5. Experiments conducted under varied conditions—three laser power levels (250 W, 300 W, 350 W), three scan speeds (6.35 mm/s, 8.47 mm/s, 10.58 mm/s), and a wide range of powder feed rates (4.93 g/min to 17.95 g/min)—revealed that process parameters significantly affect surface texture and defect formation. These observations reinforce the importance of optimizing DED parameters to balance surface quality and mitigate defects [148]. Investigations showed surface defects like the balling phenomenon occur due to low heat input resulting from a combination of low current (50 A), the designated scan speed, and a feed rate of λm = 0.0293 g/m. As the current was further reduced from 250 A to 50 A, the clad size increased, leading to higher energy densities, with the maximum value triggering the balling effect. This behavior aligns with previously reported findings [151,152]. Based on the tested parameter range, it can be inferred that an energy density (E_v_, clad) greater than 49.43 J/mm^3^ serves as the threshold for the occurrence of balling phenomena [148]. While polished and etched 316L stainless steel samples highlight variations in surface roughness, surface analysis reveals variation in roughness, with smoother areas observed in regions where particle incorporation was more complete. In contrast, areas with increased roughness are attributed to partially incorporated particles on the clad surface. These defects likely result from the formation of larger powder islands caused by insufficient energy input at high powder feed rates, which hinders full integration of particles into the melt pool [148].

In a study, the influence of laser power, scan speed, hatch spacing, and energy density on surface roughness and mechanical properties was systematically analyzed for 316L stainless steel fabricated using selective laser melting (SLM). The research examined how variations in these key process parameters affected the alloy’s surface characteristics and overall structural performance. Table 8 presents the surface roughness corresponding to different SLM processing conditions [143].

**Table 8:** The table provides a summary of the impact of essential process parameters, such as laser power, scan speed, hatch spacing, and energy density, on the surface roughness of 316L stainless steel produced through selective laser melting (SLM). The findings emphasize the importance of optimizing these parameters to enhance surface quality and structural performance [143].

Sample No	Laser Power (P) [W]	Scan Speed (V) [mm/s]	Hatch Spacing (h) [mm]	Energy Density (E) [J/mm^3^]	Surface Roughness (Ra) [µm]
1	170	1000	0.08	53.13	4.51
2	170	1050	0.1	40.48	5.64
3	170	1100	0.12	32.2	6.47
4	195	1000	0.1	48.75	4.07
5	195	1050	0.12	38.69	6.05
6	195	1100	0.08	55.4	5.32
7	220	1000	0.12	45.83	4.89
8	220	1050	0.08	65.48	4.3
9	220	1100	0.1	50.0	5.32

Continuing the analysis, Table 9 presents surface roughness measurements (R_a,x_ and R_a,y_) along with detailed process descriptions for samples produced using laser powder bed fusion (L-PBF) such as SLM, directed energy deposition only (DEDo), and directed energy deposition with laser scanning (DED + LS). These measurements highlight the significant influence of various processing parameters and post-processing techniques—such as milling, grinding, and feed speed—on surface quality and treatment outcomes. The table also provides corresponding codes and descriptions for better clarity. By comparing these methods, the data underscores the critical role of parameter optimization in achieving smoother surfaces and enhancing part performance. This finding aligns with previous discussions on 316L stainless steel alloys fabricated using DED techniques, further reinforcing the interdependence between process conditions, surface roughness, and the final properties of manufactured components [149].

Building on the previous discussion, a separate study compared the surface roughness parameters and lattice strain values of as-built and ground samples to evaluate the impact of post-processing techniques on surface quality and internal stresses. Table 10 presents the measured values for both conditions, highlighting significant differences in surface characteristics, including roughness parameters and lattice strain across various crystal planes. This comparison underscores the critical role of surface modification methods, such as grinding, in achieving smoother surfaces, reducing residual stresses, and enhancing the functional performance of additively manufactured components, particularly in applications demanding precise surface properties and improved mechanical behavior [154].

### 3.3. Residual Stresses

Residual stresses remain a major challenge in selective laser melting (SLM) processes. The cited study investigates strategies to identify residual stress amounts in 316L stainless steel by employing both preheating and in situ rescanning techniques. Preheating was applied at temperatures of 100 °C, 200 °C, 300 °C, and 400 °C, effectively reducing cooling rates through diminished thermal gradients and elevating the maximum melt pool temperature. Notably, both baseplate and powder bed preheating yielded similar outcomes, with no significant differences in their effects on melt pool temperatures or cooling rates. Higher preheating temperatures, particularly at 400 °C, nearly eliminated residual stresses—especially at the center of the top surface. In contrast, while rescanning also contributed to a reduction in residual stresses, its impact was less pronounced and was strongly dependent on the laser power, with the optimal reduction occurring when the rescanning laser power matched the scanning power. These findings highlight the potential of optimized preheating and rescanning protocols for minimizing residual stresses in SLM-fabricated components [120].

Residual stresses in 316L stainless steel fabricated through directed energy deposition (DED) were evaluated using the semi-destructive hole-drilling strain gauge method. Cubic samples (30 mm × 30 mm × 30 mm) were produced with two different scanning strategies: 0–90° and 0–67° rotation per layer. Residual stress measurements were taken on the top surface and lateral sides (Side A and Side B) to a depth of 2 mm. The 0–90° scanning strategy generated higher residual stresses, particularly on the lateral surfaces, due to steeper thermal gradients caused by frequent directional shifts. In contrast, the 0–67° strategy minimized thermal mismatches, resulting in lower residual stresses. These findings, as summarized in Table 11, emphasize the impact of scanning strategies and cooling rates on residual stress distribution [135].

### 3.4. Comparison Analysis of Defects

The comparative analysis of defect formation in directed energy deposition (DED), electron beam melting (EBM), and selective laser melting (SLM) reveals significant variations in defect types. Each process presents distinct challenges—such as porosity in DED, cracking in EBM due to strain aging, and residual stress accumulation in SLM. These differences notably affect the mechanical properties, surface quality, and overall structural integrity of the manufactured components, as detailed in Table 12.

SLM provides the best balance of mechanical strength, density, and precision but is highly prone to residual stress accumulation, porosity, and warping, requiring additional stress-relief treatments. DED, while suitable for large-scale manufacturing and repair, suffers from poor surface finish, higher porosity, and inconsistent layer bonding, limiting its use in high-performance applications. EBM effectively minimizes residual stresses and oxidation-related defects but exhibits coarser microstructures and lower resolution, reducing its suitability for intricate components. For applications that demand high strength, fine resolution, and structural integrity, SLM remains the most viable option, provided that stress management techniques and process optimizations are implemented. DED is preferable for cost-sensitive, large-scale applications, while EBM is the best choice for stress-sensitive and oxidation-resistant components [143,153,155,156,157].

## 4. Post-Processing

**Figure 6:**
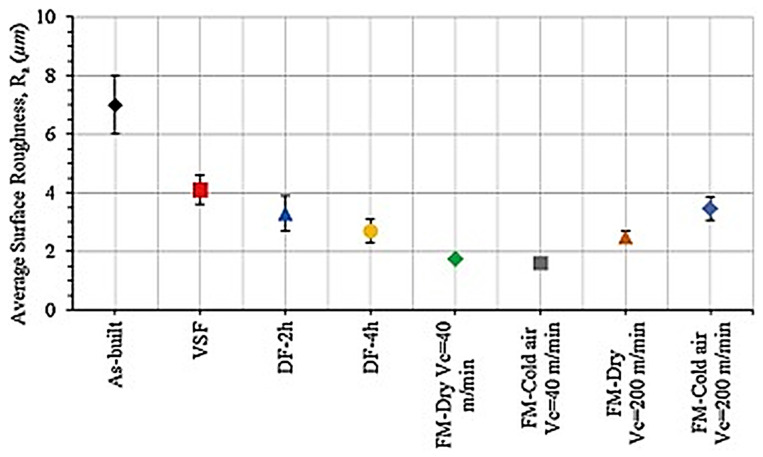
In the SLM of 316L stainless steel, post-processing methods such as finish machining (FM), vibratory surface finishing (VSF), and drag finishing (DF) were evaluated for their influence on surface roughness, with drag finishing demonstrating the most significant impact [158]. Sourced from publisher with permission.

Several post-processing techniques (discussed in this section) are essential for enhancing additively manufactured 316/316L stainless steel components. These methods play a critical role in reducing surface roughness, porosity, and residual stresses while promoting a more uniform microstructure and lowering the risk of cracks and other surface defects. Figure 6 illustrates the impact of finish machining (FM), vibratory surface finishing (VSF), and drag finishing (DF) on the surface roughness of SLM-fabricated 316L stainless steel, with drag finishing demonstrating the most significant improvement.

Surface roughness characterization of additively manufactured 316L stainless steel was performed using white light interferometry across multiple build orientations, including side, top, up-skin, and down-skin surfaces. These surfaces underwent various post-processing treatments—namely as-printed, electro-polished, tumble-polished, and re-melted via contour scanning. Quantitative roughness metrics, such as average surface roughness (S_a_) and peak-to-valley height (S_z_) were reported for each condition. The data, presented with standard deviation error bars and individual measurement points, highlight how surface texture evolves with both build orientation and post-processing method. The plots results (surface roughness measurements) of the cited study illustrate these trends, emphasizing the comparative effects of each treatment on surface quality across different regions of the printed components [159].

In addition, polishing, peening, machining, and finishing play a crucial role in controlling defects in additively manufactured materials. Table 13 outlines the impact of various post-processing techniques on surface quality, defect reduction, and property enhancements of 316 and 316L stainless steel alloys. Each method is described in terms of its mechanism, specific effects on defect mitigation and surface roughness, and its overall influence on the material’s functional properties [160]. Laser-based post-processing techniques such as laser peening and laser polishing also play a crucial role in improving AM parts. Laser peening generates localized compressive stresses through plastic deformation perpendicular to the surface, enhancing fatigue resistance and mechanical properties [161,162]. Laser polishing reduces surface roughness by heating surface peaks to the melting temperature and redistributing molten material due to gravity and surface tension. Rapid cooling solidifies the surface, creating a smoother finish [163,164,165]. In addition, conventional machining and abrasive finishing techniques, widely used for their reliability and accessibility, complement these advanced methods by achieving high surface accuracy. Similarly, heat treatments such as annealing (furnace cool), normalizing (air cool), and quenching (water/oil cool) are commonly employed to reduce residual stresses and enhance mechanical properties [166,167].

The effects of shot peening on the residual stress and mechanical properties of 316L stainless steel produced through selective laser melting (SLM) in comparison to conventionally manufactured (REF) samples are illustrated in Table 14. It assesses how multiple shot peening passes influence residual stress distribution, particularly at the surface and 200 µm depth, while also analyzing changes in ultimate tensile strength (UTS), yield strength (YS), hardness, and ductility [168].

Moreover, researchers have consistently shown that the mechanical properties and microstructures of additively manufactured (AM) materials are significantly influenced by the choice of post-processing techniques [169,170,171,172,173,174,175,176,177,178]. One widely used method is hot isostatic pressing (HIP), which employs high pressures (up to 200 MPa) and high temperatures (typically 1000–2000 °C) using inert gas media. This technique applies uniform pressure in all directions, eliminating inherent defects and pores in powder bed fusion (PBF) processes to yield parts with superior density, mechanical performance, and reliability, while also reducing production cycles and conserving energy [179,180]. For example, in selective laser melting (SLM)-fabricated 316L stainless steel, a heat treatment at 450 °C (SLM-450) has been found to improve corrosion resistance and relieve stress, largely due to the formation of MnSiO_3_ phases that mitigate toxic cation (Ni^+2^, Cr^+3^, Mo^+6^ etc) concentrations. In contrast, heat treatments in the 650–1050 °C range have a pronounced impact on creep performance. At lower temperatures (around 650 °C), the refinement of dislocations enhances creep life, whereas at higher temperatures (approximately 900 °C), the coarsening of dislocation cells diminishes creep resistance. Recrystallization at 1050 °C produces equiaxed grains, eliminating solute (particularly of Cr and Mo at dislocation cell boundaries) segregation and enhancing structural uniformity, thus highlighting the critical role of dislocation cells and subgrains in strengthening mechanisms [181,182]. SLM-fabricated 316L samples subjected to a 1 h heat treatment at 1050 °C—followed by air cooling or quenching—exhibit dissolution of melt pool boundaries and increased grain sizes; air-cooled samples show a 12.5% increase, while quenched samples display up to a 50% increase. These cooling methods improve ductility and reduce hardness, with an increase in strength compared to as-built samples. In addition, heat treatments at 400 °C and 650 °C tend to increase porosity and grain size, whereas tempering at 1100 °C leads to the formation of MnCr₂O₄ inclusions that further alter the microstructure. Notably, SLM samples treated with HIP at 1100 °C and 100 MPa for 1.5 h demonstrate reduced porosity, eliminated cellular structures, and decreased dislocation densities. Although these treatments significantly enhance elongation, they can reduce wear resistance due to surface softening; nonetheless, untreated SLM samples generally exhibit wear resistance comparable to conventionally manufactured stainless steel [183,184]. Similarly, SLM-manufactured 316L stainless steel components exhibit tensile residual stress near the surface and compressive stress in the core. Heat treatments at 400 °C and 650 °C achieved moderate stress relief of 24% and 65%, while solution annealing at 1100 °C achieved ~90% stress relief within 5 min, with no further improvement from longer holding times. The stress relief is attributed to microstructural changes, such as dislocation reduction, and its impact on mechanical properties is explored. In Table 15, the residual stress responses are presented along with the corresponding heat-treated conditions [25].

In a detailed study, a solution annealing process was performed at 1100 °C for 0.5 h in an atmosphere of 100 sccm (Standard Cubic Centimetres per Minute) argon and 10 sccm hydrogen, followed by furnace cooling. When combined with HIP, this treatment produced SLM 316L stainless steel with a hardness of 230.5 HV, an ultimate tensile strength of 733 MPa, a yield strength of 512 MPa, and a maximum elongation of 70.1%. This solution treatment notably enhanced ductility and overall mechanical performance in hybrid samples [184]. Complementary annealing studies have revealed that 316L remains phase stable up to 873 K (599.85 °C) with fine subgrains and cellular structures persisting; however, at higher temperatures grain coarsening reduces strength. Heat treatments in the 400–1100 °C range can relieve up to 90% of residual stress while inducing significant microstructural changes, such as the formation of an embrittling σ-phase between 650 and 800 °C, which distinguishes AM 316L from its wrought counterpart [25,185]. In comparison, electron beam melting (EBM)-fabricated 316L generally requires less heat treatment due to its inherently favorable mechanical properties, though surface finishing may still be necessary for specific applications [97,184].

The effects of heat treatments are also pronounced in 316L stainless steel fabricated via direct laser deposition (DLD). For instance, air cooling after two hours of furnace heating promotes grain growth, eliminates interlayer porosity, and removes laser track footprints. Furthermore, such heat treatments transform low-angle grain boundaries into high-angle ones, enhancing twinning and activating additional slip systems. These microstructural changes lead to the formation of a twinned austenite phase with a face-centered cubic (FCC) structure and a reduction in the δ-ferrite phase, thereby improving overall material properties [153]. Quenching from 1273 K reduces ferrite content and promotes columnar grain growth; although this decreases hardness by about 5.47% due to grain coarsening, it results in lower yield and tensile strengths (reductions of 17% and 5%, respectively) while increasing ductility by 26%—illustrating the inherent trade-offs in thermal treatments [115,153,186,187,188]. Similarly, studies on laser powder directed energy deposition (LP-DED) have shown that stress relief (SR), solution annealing (SA), and HIP yield varied outcomes. SR at temperatures below 1000 °C retains melt pool boundaries and fine cellular structures due to limited diffusion, while treatments above 1000 °C induce partial recrystallization, increased grain size, and annealing twins. As-built samples typically exhibit the highest strength but lower elongation, emphasizing the strength–ductility trade-off inherent to these processes [189]. Combined HIP and precipitation annealing effectively remove porosity and melt pool boundaries while promoting grain growth; however, this approach may introduce residual stresses, increasing stress levels by 30–40%, whereas precipitation annealing alone can increase porosity and induce more pronounced plastic cracking in high-energy-density samples [190].

Table 16 presents a comprehensive comparison between additively manufactured (AM) and conventionally manufactured stainless steel alloys, focusing on various post-processing techniques applied to 316 and 316L stainless steels. It highlights the processing conditions, material orientation, and the resulting enhancements in mechanical properties. It details how different post-processing methods influence yield strength (YS), ultimate tensile strength (UTS), and elongation (ε %), providing insight into their effectiveness in refining microstructure, reducing defects, and improving overall mechanical performance. The reference column indicates the studies that have contributed to these findings, allowing for a comparative analysis of post-processing strategies used to optimize additively manufactured materials.

### 4.1. Post-Processing Effects on Microstructure

During heat treatment of hybrid additive–subtractive process HASP-fabricated 316L stainless steel, the σ- and δ-phases progressively diminish. The heat treatment process for HASP 316L samples includes (a) untreated HASP; (b) heating at 950 °C for 3 min followed by water quenching (WQ); (c) heating at 1000 °C for 3 min with WQ; (d) heating at 1050 °C for 3 min; (e) heating at 1150 °C for 3 min with WQ; and (f) heating at 1150 °C for 30 min followed by WQ. With increased temperature and prolonged holding, such as at 1150 °C for 30 min, a fully austenitic structure emerges as these phases completely dissolve. In as-built samples, δ-ferrite formation is influenced by alloying elements like Mo, Cr, and Si, which promote its presence during rapid solidification. In the as-built condition, the microstructure is composed of δ-ferrite and σ-phases embedded within a γ-austenite matrix, resulting from rapid solidification and thermal gradients during the DED process. As heat treatment is applied, the microstructure evolves significantly. At 950 °C and 1000 °C, the σ-phase starts dissolving, while the δ-phase transitions into finer structures. By 1050 °C the σ-phase is nearly eliminated, and the δ-phase shows further transformation. At 1150 °C for 3 min, the σ-phase is entirely dissolved, leaving a nearly uniform austenitic structure. Prolonging the treatment to 30 min at 1150 °C results in a fully austenitic structure with larger grains due to extended heat exposure. These changes highlight the influence of temperature and duration in refining the microstructure and improving material properties [194].

In the as-built condition of 316L SS in the case of SLM and DED, the remaining phase is predominantly austenite, with an average ferrite content of approximately 1.4%. However, during heat treatments at 650 °C for 2 h (HT1), 650 °C for 6 h (HT2), 1150 °C for 2 h (HT3), and 1150 °C for 4 h +  1066 °C for 1 h, the ferrite content significantly decreased, with successive values recorded as 0.9%, 0.8%, and 0.1%, respectively. This reduction can likely result in spinodal decomposition, transforming into Cr-rich alpha and Fe-rich alpha phases [195]. Additionally, ferrite serves as a supportive matrix for precipitation, promoting the nucleation of M_23_C_6_ carbides. Annealing 316L stainless steel at 1050 °C can mitigate the presence of ferrite by dissolving it and preventing secondary precipitation. As the temperature increases, the dissolution kinetics of the ferrite phase accelerate. The microstructures of SLM and DED samples in both the as-built condition and after heat treatment (1150 °C for 4 h  +  1066 °C for 1 h), along with the corresponding heat treatment parameters [115,196], show columnar grains in PBF (SLM) and a mix of epitaxial and equiaxed grains in DED. After heat treatment, the microstructure transitions to more equiaxed grains in DED, while PBF retains columnar grains. Heat treatment conditions are detailed for each state, highlighting the evolution in grain morphology [194].

**Figure 7:**
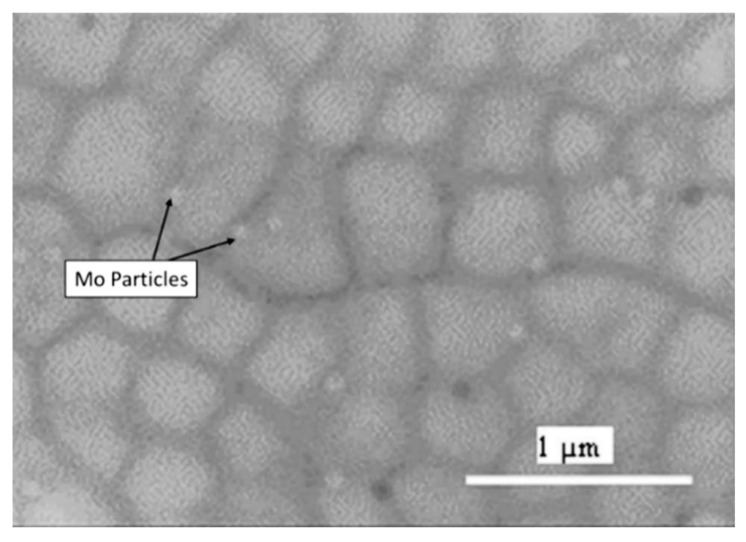
Microstructure of EBM-Fabricated 316L stainless steel highlighting Mo-rich precipitates. Image used with permission from publisher [86].

EBM-fabricated 316L stainless steel, as analyzed through XRD and EBSD, predominantly exhibits an FCC austenitic phase (99.5%) with minor traces of ferrite and cementite (shown in Figure 7). A slight reduction in molybdenum (Mo) content is observed due to its high evaporation rate during processing. The resulting microstructural variations—driven by melt pool dynamics, rapid solidification, and temperature gradients—lead to a transformation from cellular to columnar grain structures. Chromium- and molybdenum-rich precipitates are observed along grain boundaries, where Mo agglomeration significantly influences phase stability and local chemical composition. Despite achieving over 99% density, occasional crescent-shaped pores appear, attributed to localized energy input deficiencies [195].

**Figure 8:**
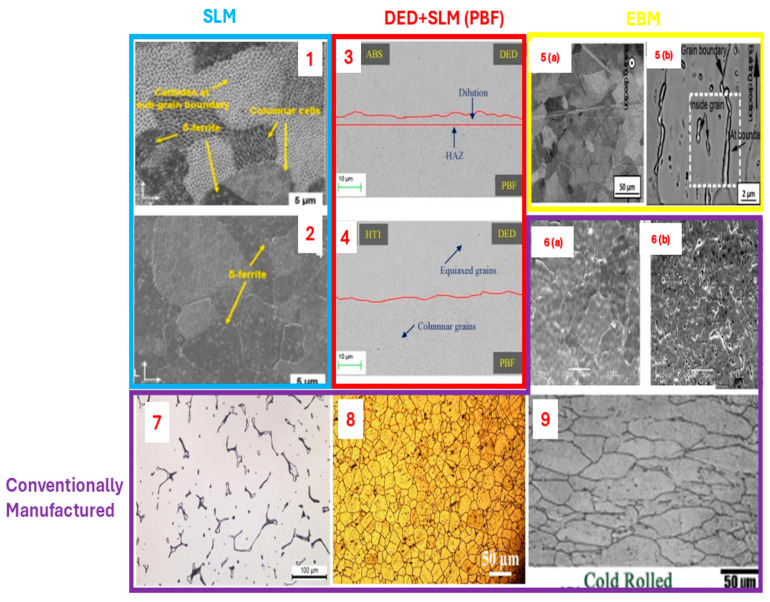
The microstructures illustrations of additively manufactured Samples: {(**1**) SLM as built, (**2**) SLM+HIP}, {(**3**) DED and SLM sandwich type sample as built; (**4**) DED and SLM sandwich sample heat-treated at 650 °C furnace cooled [197]}, {EBM as-built where, (**5a**) shows EDS-mapped precipitates within grains and at boundaries and (**5b**) shows severe localized precipitation observed in specific regions [39]}. Conventionally manufactured sample microstructures: {(**6**) PIM-processed where, (**6a**) is sintered in Hydrogen atmosphere and, (**6b**) sintered in a vacuum} [199], (**7**) as-cast [203], (**8**) cold forged [204], and (**9**) cold rolled [205]}. Micrographs of SLM, DED, and EBM are taken from the cited reference and have been reproduced with permission from the publisher. Micrographs were validated and identified by comparing the cited studies, and the studies showed quite similar micrographs [39,184,196,198,199,200,201,202].

Figure 8 presents a comparative analysis of the microstructures of 316/316L stainless steel fabricated using selective laser melting (SLM), directed energy deposition (DED), and electron beam melting (EBM), in both as-built and post-processed conditions, alongside conventional manufacturing methods such as powder injection molding, casting, forging, and cold rolling. These are compared with the microstructure of conventionally manufactured 316/316L stainless steel. The investigation highlights the impact of additive manufacturing and subsequent post-processing on grain morphology, phase distribution, and precipitation behavior, offering insights into how these methods influence the material’s mechanical and functional properties. The as-built structure produced by selective laser melting (SLM) shows a cellular microstructure composed of δ-ferrite, with carbides decorating the sub grain boundaries [184]. In the as-built condition, the 316L stainless steel fabricated by sandwich techniques using DED and powder bed fusion as SLM exhibits a predominantly austenitic microstructure, characterized by fine cellular and columnar grains aligned along the build direction. In contrast, the directed energy deposition (DED) region shows a coarser austenitic structure with a mixture of equiaxed and columnar grains, often exhibiting epitaxial growth due to remelting between layers. Localized grain refinement is also observed at the interface, influenced by the thermal gradient and overlapping thermal cycles from the two processes. Upon applying heat treatments, distinct microstructural evolutions are observed. The HT1 treatment, involving stress relief at 650 °C for 2 h followed by furnace cooling, results in a reduction in residual stresses and the formation of annealing twins within the austenitic matrix. This treatment maintains the overall grain structure while enhancing microhardness. Conversely, the HT2 treatment, consisting of solution annealing at 1100 °C for 2 h followed by furnace cooling, leads to significant grain growth and the formation of grain boundary faceting. This high-temperature exposure reduces microhardness but substantially increases ductility, as evidenced by a total elongation of 72.5% [196,197]. Electron beam melting (EBM) yields a microstructure that is almost entirely composed of the FCC austenitic phase (99.5%), with only minor traces of ferrite and cementite, while precipitates are observed within the grains and along their boundaries [39,198]. Additionally, when SLM-fabricated 316L stainless steel is subjected to hot isostatic pressing (HIP), a small number of carbides and δ-ferrites remain, although these phases gradually dissolve into the matrix with extended heat treatments [184]. In comparison to conventional manufacturing, powder injection molding (PIM) shows that the sintering atmosphere plays a crucial role: sintering in hydrogen produces a single austenite phase, whereas vacuum sintering results in an austenitic matrix embedded with M_23_C_6_ carbides [199]. In the as-cast condition, 316L stainless steel reveals a vermicular δ-ferrite pattern with straight-sided segments that suggest a specific orientation relationship between δ-ferrite and γ-austenite, as confirmed by light microscopy [200]. The microstructure of 316 stainless steel processed by 15 passes of forging is marked by severe plastic deformation zones and slip bands, with chromium carbide precipitates present in a matrix dominated by both martensitic and austenitic phases [201]. Finally, when 316L stainless steel is subjected to 70% cold rolling, the equiaxed austenite grains become flattened into a pancaked structure due to plastic deformation, and XRD analysis confirms the emergence of a martensitic phase [202].

Table 17 provides a detailed comparison of the microstructural characteristics of 316 stainless steel fabricated using various advanced manufacturing techniques, including SLM, DED, and EBM, under both as-built and post-processed conditions. It highlights the influence of heat treatment and processing conditions on grain size, dislocation structures, phase composition, and other microstructural features. The data offer valuable insights into how manufacturing and thermal treatments affect the material’s properties compared to conventional manufacturing methods.

### 4.2. Post-Processing and Precision

A precision-engineering-focused post-processing strategy for 316L stainless steel parts produced via directed energy deposition (DED), combining milling, grinding, and magnetic field-assisted finishing (MAF) within a machining center has been studied for the tool industry [206]. The approach prioritizes achieving superior dimensional accuracy, surface quality, and functionality while preserving the material’s microstructure and hardness. Grinding refines the surface by removing milling marks and introducing compressive residual stresses, optimizing the pre-polishing stage to reduce polishing time. Integrating post-processing steps such as milling or grinding before magnetic abrasive finishing (MAF) significantly improves process performance in DED-fabricated 316L stainless steel by reducing total specific energy from approximately 500 to 250 J/mm^3^, lowering MAF energy consumption to between 4000 and 12,000 J/mm^3^ depending on the feed rate, and enhancing material removal efficiency in the range of 0.26 to 0.5 mm^3^/min. This method leverages one-time chucking to streamline operations, ensuring minimal deviation and repeatability, aligning with precision engineering goals for high-performance, reliable, and geometrically accurate additive-manufactured components. Surface roughness (R_a_) outcomes were assessed for 316L stainless steel components produced via directed energy deposition (DED) and subsequently finished using various grinding and polishing tools. Tools arranged in lateral configurations (1L, 2L, and 3L) resulted in relatively high roughness values, ranging from 3.9 to 4.3 µm Ra. In contrast, conventional lateral tools (4L and 5L) demonstrated improved surface quality, achieving R_a_ values of 1.0 and 0.7 µm, respectively. Notably, vertical tool configurations (2V, 6V, 7V, and 8V) yielded superior finishes, with tool 8V achieving the lowest surface roughness at just 0.1 µm Ra. These findings underscore the critical role of tool geometry, orientation, and selection in post-processing, as vertical configurations consistently outperformed lateral ones. The study highlights the potential benefits of integrating advanced finishing tools within machining centers to achieve the fine surface finishes required for high-precision applications [206]. However, these processes (grinding, polishing, machining, etc.) are often time-consuming and expensive, as they require specialized equipment, skilled operators, and extended machining time. This can limit their practicality for large-scale or cost-sensitive production environments, highlighting the need to balance surface quality with economic and operational efficiency [207,208,209].

Directed energy deposition (DED) enables the production of near-net shape components; however, post-processing is necessary to enhance surface quality and ensure dimensional accuracy. Selective laser melting (SLM) excels in producing intricate geometries but faces challenges with residual stresses and porosity. Electron beam melting (EBM) ensures excellent material density but often necessitates additional finishing for enhanced surface accuracy. Post-processing methods like milling, grinding, and magnetic abrasive finishing (MAF) are crucial to refining the precision engineering, surface, and functionality of additive-manufactured parts. Additionally, the study examines the machining and surface integrity of 316L stainless steel produced via selective laser melting and electron beam melting for precision engineering applications. SLM parts exhibit finer as-printed surface roughness (~6 µm) compared to EBM (~41 µm), but both require machining to meet functional requirements. Post-machining, surface roughness improves to ~1.5 µm for SLM and ~1 µm for EBM under optimized parameters. Residual stress profiles differ significantly, with SLM producing tensile stresses (~570 MPa), while EBM generates beneficial compressive stresses (~150 MPa). Machining transitions these stresses to a combination of tensile at the surface and compressive below (~30 µm depth). Work hardening is more pronounced in EBM, achieving ~500 HV0.1 hardness compared to ~400 HV0.1 for SLM. Additionally, SLM showed negligible tool wear during machining, while EBM induced greater wear at higher cutting speeds (up to 0.03 mm). These findings highlight that SLM is suited for fine surface finishes and minimal wear, whereas EBM excels in applications requiring high compressive stresses and durability. The research underscores the importance of process optimization in additive manufacturing and machining to enhance surface integrity and performance in precision components [210].

## 5. Limitations of Current Review and Future Advancements

Based on the discussion presented in this paper, key limitations and research gaps in the current literature on the additive manufacturing of 316/316L stainless steel have been identified and highlighted. Despite significant advancements in understanding and optimizing additive manufacturing (AM) processes for 316/316L stainless steel, the existing literature presents several critical limitations that constrain the development of universally applicable standards and reproducible outcomes. A key issue is the lack of parameter standardization across studies. Numerous investigations report varying ranges for essential parameters such as laser power, scanning speed, hatch spacing, preheating temperature, and layer thickness. While these variations are often necessary to accommodate specific machine configurations or material batches, they hinder direct comparison and consolidation of findings. For instance, SLM parameters affecting tensile strength and porosity often differ even when using the same alloy, making it difficult to establish universally accepted optimization windows.

Closely related to this is the challenge of reproducibility. Many studies (as discussed above) report mechanical property improvements based on isolated experimental conditions without clear justification for parameter selection or validation through repeated trials. For example, reports of ductility improvements under specific build orientations or preheating conditions in DED or EBM are often based on limited sample sizes or single build directions, limiting their generalizability. Furthermore, the influence of microstructural heterogeneity such as the formation of δ-ferrite, sigma phases, or carbides is frequently observed but not consistently correlated with the process parameters or environmental conditions that induce them, pointing to gaps in thermodynamic and kinetic modeling during AM processing.

Another notable limitation is the inconsistent reporting of process metadata, including shielding gas composition and flow rates, beam spot sizes, scan rotation strategies, and energy density calculation methods. Such omissions restrict the ability to replicate findings or integrate them into machine-learning-driven parameter optimization frameworks. While some studies detail specific preheating conditions or scan strategies, others fail to specify even fundamental variables such as layer thickness or build orientation, which are known to significantly impact residual stress and surface roughness.

Additionally, the current literature often lacks integration between in situ monitoring and post-process validation, resulting in a gap between real-time control and post-build quality assurance. Although recent work highlights promising developments in real-time strain sensing and AI-based predictive models for structural monitoring, these are rarely adopted in parametric optimization studies, limiting their practical impact.

Finally, while the majority of studies (as discussed throughout this paper) focus on mechanical properties like tensile strength and hardness, fewer explore long-term performance metrics such as fatigue life, corrosion resistance, or structural stability under thermal cycling, particularly for components deployed in harsh environments. This narrow focus results in optimization strategies that may achieve high strength but overlook durability, potentially compromising application performance.

Collectively, these limitations underscore the need for more comprehensive, standardized, and reproducible experimental frameworks, ideally supported by robust in situ diagnostics and post-process validation techniques. Bridging these gaps will be essential to advancing AM from a promising fabrication technique to a fully mature, industrially deployable manufacturing solution for 316/316L stainless steel components.

- Based on the analysis presented in this paper, future advancements in additive manufacturing (AM) of 316/316L stainless steel must address a range of persistent technical and industrial challenges to realize the full potential of this technology. These include overcoming issues related to residual stress, porosity, anisotropy, and surface quality, all of which significantly influence the mechanical properties and long-term performance of printed components. As demonstrated in the comparative evaluation of DED, SLM, and EBM techniques, each method presents specific strengths and limitations, but none offer a fully optimized solution across all performance metrics. Hence, advancements must focus on both refining existing processes and integrating multidisciplinary innovations.
- One critical area for advancement lies in enhanced process optimization. The mechanical properties and integrity of AM parts are highly sensitive to processing parameters such as energy density, scan speed, layer thickness, and preheating temperature. Precise calibration and dynamic adjustment of these parameters, potentially guided by artificial intelligence or machine learning algorithms, can help in reducing residual stresses and improving build reliability. In particular, maintaining consistent thermal gradients and minimizing cooling-induced defects are key to achieving isotropic mechanical behavior and reliable microstructural formation. Such optimization strategies should be developed not only at the parameter level but also in conjunction with machine architecture and build environment control.
- Another essential future direction is the integration of advanced post-processing techniques. As shown in this study, post-processing steps such as heat treatment, hot isostatic pressing (HIP), stress-relief annealing, and various surface finishing operations play a pivotal role in mitigating microstructural inconsistencies and enhancing surface properties. Future advancements should focus on tailoring post-processing protocols to specific AM processes and material grades. For instance, optimizing the sequence and duration of heat treatment cycles based on build orientation and thermal history could result in significantly improved tensile strength, ductility, and corrosion resistance. Hybrid manufacturing workflows that combine additive and subtractive methods, such as grinding and magnetic abrasive finishing (MAF), are particularly promising in achieving dimensional accuracy and surface finish without compromising material integrity.
- Future studies should focus on integrating thermodynamic simulations with in situ thermal monitoring to achieve more accurate control over phase transformations during additive manufacturing. By coupling real-time thermal data with predictive modeling frameworks, such as calculation of phase diagram (CALPHAD)-based or phase-field simulations, it becomes possible to anticipate microstructural evolution dynamically. This integration would enable on-the-fly process adjustments to mitigate undesirable phases and ensure targeted mechanical performance. Additionally, leveraging AI to interpret thermal profiles could enhance predictive fidelity and facilitate the development of closed-loop systems for microstructure control.
- A third area requiring focused innovation is the alignment of AM with industrial requirements. While laboratory results are encouraging, scalability and standardization remain major hurdles. Applications in aerospace, biomedical, and energy sectors demand not only high-performance materials but also rigorous quality control and certification. Therefore, future advancements should target the development of standardized protocols for material qualification, mechanical testing, and defect assessment. This includes expanding databases for mechanical and corrosion properties of AM 316L components under various loading and environmental conditions. Furthermore, the integration of AM within digital manufacturing ecosystems—using digital twins, real-time quality monitoring, and closed-loop feedback systems—will be essential for consistent and repeatable part production.
- In situ monitoring and computational modeling represent another frontier for future growth. The ability to monitor the build process in real time—through thermal imaging, melt pool sensors, or acoustic feedback—can significantly improve defect detection and reduce material waste. Coupling such monitoring systems with computational models allows for predictive control over the process and facilitates proactive adjustments. For example, thermomechanical simulations can forecast residual stress buildup, while microstructure evolution models can anticipate phase transformations and grain growth patterns. These tools will help bridge the gap between theoretical material science and practical AM engineering.
- Lastly, sustainability considerations will become increasingly central to future AM strategies. As industries push toward environmentally responsible manufacturing, AM must reduce its energy footprint, optimize powder usage, and enable recycling pathways. Research into energy-efficient beam sources, reusable support structures, and life-cycle assessments of AM parts will support the transition to greener manufacturing.
- In summary, the future of additive manufacturing for 316/316L stainless steel lies in a comprehensive strategy that integrates process refinement, smart monitoring, post-processing innovation, and industry-aligned qualification standards. The convergence of materials science, digital engineering, and sustainability research will drive the evolution of AM from a specialized technique into a mainstream manufacturing solution. Ultimately, for future trends—such as real-time quality monitoring, AI-driven process control, and alloy design tailored for additive processes—the alignment of fundamental scientific research with practical industry requirements forms the cornerstone of advancing AM techniques.

## 6. Conclusions

- This comprehensive review provides an in-depth and scientifically rigorous evaluation of additive manufacturing (AM) processes—specifically directed energy deposition (DED), selective laser melting (SLM), and electron beam melting (EBM)—and their capability to optimize process parameters, the microstructural integrity, mechanical performance, and defects characteristics of 316/316L stainless steel.
- A key takeaway highlighted in this work is the clear but complex interdependency between AM process parameters—such as energy density, laser power, scanning strategy, layer thickness, and preheating conditions—and the resulting mechanical properties of 316/316L stainless steel. This understanding is pivotal for aligning manufacturing practices with industry-specific performance requirements, especially within high-precision sectors such as aerospace, biomedical implants, and automotive engineering.
- This comparative evaluation elucidates the strengths and limitations inherent in each AM methodology. SLM is identified as the most promising technique in terms of achieving intricate geometries and superior microstructural refinement; however, its widespread industrial adoption is constrained by significant residual stress accumulation and porosity, mandating sophisticated parameter control and extensive post-processing. In contrast, DED provides unparalleled flexibility for large-scale component production and in situ repairs but requires considerable attention to residual stress reduction, surface roughness, and uniform property distribution. EBM distinctly offers effective management of thermal stresses through its high operational temperatures and vacuum environment, although its broader applicability is hindered by constraints in material compatibility, feature resolution, and operational cost.
- This review notably emphasizes alignment between AM processing parameters and defect mitigation strategies, clearly delineating pathways such as process optimization, defect monitoring/inspection, and post-processing treatments toward achieving consistent quality and performance reliability. Advanced post-processing techniques, particularly hot isostatic pressing (HIP) and heat treatments, are presented as essential alignment tools to refine microstructures, minimize defects, and enhance material performance for demanding environments. Scientifically, this review underscores the necessity for developing comprehensive and optimized AM parameters to mitigate AM-induced anisotropy and residual stresses, promoting enhanced predictability and reproducibility of 316/316L stainless steel components.
- Ultimately, for future trends such as AI, ML, CALPHAD, simulations, and real-time monitoring, the alignment of fundamental scientific research with practical industry requirements forms the cornerstone of advancing AM techniques. While this review has not directly explored in situ monitoring or computational modeling, both represent promising strategies that have shown potential to improve build quality, defect detection, and process control [211,212,213]. Their limited application to AM-fabricated 316/316L stainless steel highlights a valuable opportunity for future research. Prioritizing such approaches—especially when combined with rigorous experimental validation—could significantly accelerate the industrial implementation of AM components for high-performance applications.

## Figures and Tables

**Table 2:** Comparative analysis of additive manufacturing techniques: DED, SLM, and EBM (sourced from publisher under permission) [34,35,36].

Parameters	Additive Manufacturing Techniques		
	DED	SLM	EBM
Energy Source	Laser-based system	Laser-based system	Electron beam system
Typical Power	Approximately 500 W	Around 120 W	Up to 3500 W
Beam Diameter	660–900 μm	30–250 μm	200–1000 μm
Preheating Requirement	200 °C to 500 °C	100 °C to 200 °C	Approximately 700 °C
Scan Speed	Slow (0.001–0.04 m/s)	Moderate (0.3–1 m/s)	Very fast (over 1000 m/s)
Layer Thickness	200–1000 μm	20–100 μm	50–200 μm
Post-processing Needs	Variable: stress relief may be required	Common; typically, HIP used	Minimal; due to high preheating
Mechanical Behavior	High strength with reduced ductility	Similar strength; lower ductility	Properties akin to conventional techniques
Surface Finish	Rough (20–50 µm)	Smooth (<10 µm)	Moderate (10–50 µm)
Residual Stress Levels	Significant	Significant	Low
Primary Applications	Aerospace, medical implants, industrial tooling	Precision engineering in aerospace, automotive, medical	Energy, aerospace, defense sectors

**Table 3:** Optimized DED process parameters and their impact on the mechanical properties of 316L/316 stainless steel alloy.

DED Process Parameter	Optimized Range	Primary Effects on 316/316L Stainless Steel
Laser Power (W)	~400 W	Lower laser power with high scan speed refines microstructure and improves mechanical strength
Preheating Temperature (°C)	300 °C; Cold substrate also studied	Preheating reduces residual stress and defects; cold substrate improves strength
Shielding Gas Type/Flow	Argon + 3% Nitrogen; 5–25 L/min	Proper gas mix improves strength and uniformity; poor mixtures increase porosity
Build Orientation	0° preferred over 90°	0° orientation enhances UTS and bonding: 90° increases anisotropy and interlayer weakness which affects the mechanical properties
Feed Rate (g/min)	10 g/min	Lower feed rate ensures full melting and mechanical strength; higher rates lead to porosity and defects
Deposition Pattern	Offset, Raster	Offset reduces thermal distortion; raster increases geometric flexibility
Nozzle Diameter (mm)	0.40 mm for (better strength) in comparison to other values; 0.2 mm (better consistency)	Larger diameter improves strength; smaller maintains uniformity
Infill Density (%)	15% better than 20–25%	Higher density generally increases strength, though some anomalies observed
Scan Speed (mm/min)	960–1200	Influences microstructure; minimal impact on yield strength and elongation

**Table 4:** Optimization of selective laser melting (SLM) process parameters and their impact on mechanical properties.

SLM Process Parameter	Optimized Range	Primary Effects on 316/316L Stainless Steel
Build Orientation	Choose 45° for better tensile strength and fatigue resistance; 90° for higher elongation and hardness.	Higher strength in vertical builds; better ductility in horizontal builds
Scan Rotation	Apply 45° or 67° scan rotation to increase high-angle grain boundaries and improve mechanical properties.	Increases grain boundaries, enhancing strength, toughness, and mechanical stability
Hatching Patterns	Use rectangular hatching for increased hardness; and hexagonal for better grain refinement.	Influences epitaxial grain growth, improving hardness and microstructural uniformity
Hatch Spacing	Reduce hatch spacing to enhance melt pool overlap and minimize porosity	Reduces defects, enhances microhardness, and improves part density
Scanning Speed	Select moderate scanning speed to balance tensile strength and density, avoiding incomplete melting.	Optimized speed prevents cracks, improves fusion, and enhances mechanical stability.
Layer Thickness	Maintain layer thickness between 30–50 µm for optimal microstructure and reduced defects.	Minimizes surface roughness, improves hardness, and refines grain structure
Energy Density	Keep energy density in the range of 50–125 J/mm^3^ to enhance densification and hardness.	Enhances layer bonding, reduces porosity, and prevents keyhole defects
Laser Power	Adjust laser power: lower for finer grains and hardness, higher for increased fusion and strength.	Lower power refines grains for hardness; higher power increases fusion but risks defects.
Spot Size	Use a smaller spot size for finer grains and higher resolution; larger for stability and lower porosity.	Smaller spot size enhances resolution, but risks localized porosity; larger spot reduces porosity.
Shielding Gas Flow	Set shielding gas flow between 550–600 L/min with Argon for oxidation prevention and tensile strength.	Proper gas flow minimizes oxidation, enhances tensile properties, and ensures uniform fusion.
Preheating Temperature	Preheat the build plate to ~150 °C to reduce porosity, increase ductility, and improve fatigue resistance.	Refine microstructure reduces residual stresses and improves mechanical strength.

**Table 5:** Optimized electron beam melting (EBM) process parameters and their impact on mechanical properties.

EBM Process Parameter	Optimized Range	Primary Effects on 316/316L Stainless Steel
Build Orientation	Choose vertical orientation for higher tensile strength; choose horizontal for better ductility and toughness.	Vertical builds improve strength; horizontal builds improve flexibility.
Scanning Strategy	Use 90° scan rotation to improve grain refinement, reduce residual stresses, and enhance mechanical properties.	Enhanced grain structure, reduced internal stresses, improved tensile strength, and fatigue resistance.
Preheating Temperature	Maintain preheating between 800–850 °C to minimize residual stress, reduce porosity, and improve ductility.	Better material consolidation, refined grain structure, and lower defect rates.
Hatch Spacing	Select narrower hatch spacing (0.1 mm) to enhance part density and minimize porosity; avoid wider spacing to prevent defects.	Reduces defects, increases density, improves fatigue resistance, and enhances surface quality.
Scanning Speed	Use 2800–4350 mm/s: Higher speeds minimize overheating but may reduce fusion; lower speeds improve fusion but increase defects.	Ensures proper fusion, reduces lack-of-fusion defects, and optimizes mechanical stability.
Beam Current	Set beam current to 15 mA for a balance between density, strength, and microstructural uniformity.	Maintains optimal energy input, ensuring fusion uniformity and consistent microstructure.
Layer Thickness	Use a layer thickness of 50–70 μm to maintain optimal surface finish, mechanical strength, and part density.	Provides better mechanical performance, prevents excessive roughness, and minimizes defects.
Beam Spot Size	Select a smaller beam spot size for better melt pool control and fine grain structure; use a larger spot for wider coverage but with reduced resolution.	Improves precision, reduces porosity, and enhances part resolution for high-performance applications.
Energy Density	Maintain energy density at ~64.28 J/mm^3^ to achieve maximum tensile strength, improved hardness, and reduced keyhole porosity.	Prevents microcracking, enhances part durability, and improves overall strength and hardness.
Vacuum Pressure	Increase helium (He) pressure from 10^−3^ to 10^−2^ mbar to reduce material evaporation while maintaining process efficiency.	Maintains alloy composition, reduces vaporization, and improves overall material integrity.

**Table 9:** This table provides a detailed comparison of surface roughness values (R_a,x_ and R_a,y_) for samples fabricated using different methods, including laser powder bed fusion (L-PBF), directed energy deposition only (DEDo), and directed energy deposition with laser scanning (DED + LS). Each sample code represents a specific combination of process parameters, including variations in laser power, scanning speed, and laser post-processing, with corresponding effects on surface quality.

Parameter (Code + Description)	R_a,x_ (μm)	R_a_,_y_ (μm)	R_sk_(μm)	Ref.
L-PBF: Baseline sample produced using laser powder bed fusion, without further treatment.	10.20 ± 0.74	9.80 ± 0.82	-	[149]
DEDo—1: directed energy deposition only, with high laser power and slower scanning speed.	9.00 ± 0.12	5.60 ± 0.71	-	[149]
DEDo—2: directed energy deposition only, with lower laser power compared to DEDo—1.	8.30 ± 0.65	5.60 ± 0.78	-	[149]
DED + LS—1a: directed energy deposition with laser scanning, high laser power and slower scanning.	2.00 ± 0.35	4.00 ± 0.61	-	[149]
DED + LS—1b: directed energy deposition with laser scanning, intermediate process parameters.	2.30 ± 0.42	4.30 ± 0.54	-	[149]
DED + LS—1c: directed energy deposition with laser scanning, lower laser power and moderate speed.	2.10 ± 0.48	4.00 ± 0.68	-	[149]
DED + LS—2a: directed energy deposition with laser scanning, low laser power and faster scanning speed.	2.40 ± 0.22	4.40 ± 0.32	-	[149]
DED + LS—2b: directed energy deposition with laser scanning, intermediate laser parameters.	2.40 ± 0.28	4.20 ± 0.24	-	[149]
DED + LS—2c: directed energy deposition with laser scanning, low laser power and moderate speed.	2.30 ± 0.36	4.60 ± 0.41	-	[149]
DED: Initial surface condition with high roughness.	105.39	-	-	[153]
DED + M1: Milled surface, smoother than initial, with visible tool marks.	1.55	-	0.22	[153]
DED + M1 + MAF(Vf200): Polished with MAF at a lower feed rate, reducing roughness further and improving surface smoothness.	0.39	-	−0.24	[153]
DED + M1 + MAF(Vf1012): Polished with MAF at a higher feed rate, selectively smoothing surface peaks but less effective in overall reduction.	1.1	-	−0.20	[153]
DED + M3 + G1: Ground surface with reduced roughness and removal of milling marks.	1.16	-	−0.44	[153]
DED + M3 + G1 + MAF(Vf200): Further polished with MAF at a lower feed rate, achieving smoother results.	0.67	-	−0.96	[153]
DED + M3 + G1 + MAF(Vf1012): Polished with MAF at a higher feed rate, reducing roughness but not as effectively as lower feed rates.	0.59	-	−0.52	[153]

**Table 10:** Comparison of surface roughness parameters and lattice strain in as-built and ground SLM-fabricated 316L stainless steel [154].

Parameter (Unit)	316 L-AB	316 L-G
R_a_ (Arithmetic Average Roughness, µm)	2.8 ± 0.6	0.07 ± 0.04
R_z_ (Maximum Height of Profile, µm)	9.4 ± 1.5	0.36 ± 0.04
R_pv_ (Peak-to-Valley Height, µm)	17 ± 3	0.47 ± 0.12
R_sk_ (Skewness)	−0.4 ± 0.8	−0.24 ± 0.18
R_ku_ (Kurtosis)	3.2 ± 0.7	2.7 ± 0.6
S_dr_ (Developed Surface Area Ratio, %)	7.2 ± 0.5	0.15 ± 0.02
Lattice Strain (111, %)	0.520	0.316
Lattice Strain (200, %)	0.725	0.400
Lattice Strain (220, %)	0.404	0.187
Lattice Strain (311, %)	0.406	0.235
Lattice Strain (222, %)	0.372	0.212

**Table 11:** Residual stress distribution in 316L stainless steel cubes fabricated using different scanning strategies in directed energy deposition [135].

Cube (Scanning Strategy)	Principal Stress Component	Stress Range (MPa)	Observation
(0–90°)	σ_max_	Top: −77 to 233 Side A: −50 to 635 Side B: −160 to 376	Higher cooling rates lead to higher residual stresses on lateral surfaces.
	σ_min_	Top: −164 to 165 Side A: −211 to 119 Side B: −243 to 165	
(0–67°)	σ_max_	Top: −172 to 133 Side A: −61 to 265 Side B: −179 to 278	Lower cooling rates reduce residual stresses, especially on the top surface.
	σ_min_	Top: −204 to 103 Side A: −139 to 118 Side B: −302 to 134	

**Table 12:** Comparison of defects and material properties for DED, SLM, and EBM techniques in 316L stainless steel.

Techniques	Failure Type	Defects			Refs.
		Residual Stresses	Surface Roughness (Ra)	Porosity	
DED	Formation of pores and weak metallurgical bonds	Typically exhibits tensile stresses caused by rapid cooling during manufacturing.	Ranges from 10 to 20 µm based on process parameters.	Achieves up to 99.6% density with optimal settings.	[153,155,156]
EBM	Cracking due to strain-aging effects	Lower stress levels are achieved due to elevated process temperatures.	Typically varies between 15 and 25 µm, influenced by powder size.	Porosity below 1% is achievable with careful parameter tuning.	[155,156,157]
SLM	Residual stress and fracture formation	Significant tensile stresses are common and usually require additional processing to reduce.	Falls within 5 to 15 µm depending on layer thickness and speed.	Less than 1% porosity is typically achieved when parameters are optimized.	[143,155,156,157]

**Table 13:** A detailed overview of post-processing methods including chemical polishing, electropolishing, abrasive flow machining, shot peening, laser polishing, tumbling, and ultrasonic cavitation [160].

Post-Processing Method	Mechanism/Technique	Impact on Defects	Effect on Surface Quality	Changes to Material Properties	Applications and Notes	Material
Chemical Polishing	Immersing parts in acidic or alkaline solutions, such as mixtures of phosphoric, nitric, and hydrochloric acids.	Cleans the surface by removing particles, contaminants, and debris, leaving a smooth and uniform finish.	Reduces surface roughness to approximately 5.2 μm.	Improves corrosion resistance and enhances the visual finish, making it suitable for applications requiring a polished surface.	Ideal for complex geometries, including internal channels where electrode placement is challenging.	316
Chemical Polishing	Using chemical baths to enhance smoothness and eliminate irregularities from internal and external surfaces.	Effectively removes micro-defects and imperfections in hard-to-reach areas.	Achieves roughness levels as low as 1.93 μm.	Enhances durability against environmental factors like corrosion, ensuring suitability for biomedical and industrial applications.	Particularly effective for intricate designs and parts with detailed internal features.	316L
Electropolishing	Electrochemical removal of surface layers using acids like phosphoric and sulfuric, applied under controlled current.	Smoothens external surfaces by eliminating rough patches and defects, though access to internal regions can be limited.	Reduces roughness to around 2 µm on external faces.	Increases resistance to wear and fatigue, while improving aesthetic appeal and corrosion resistance.	Best suited for external surfaces due to limitations in reaching narrow or concealed areas.	316L
Abrasive Flow Machining	Polishing by forcing abrasive-laden viscoelastic media through internal channels or over external surfaces.	Removes adhered powder and minor surface irregularities; limited effectiveness in areas where flow is restricted.	Achieves a fine finish with roughness as low as 0.4 µm.	Enhances mechanical properties like fatigue strength by minimizing stress concentrations.	Suitable for intricate geometries, such as cooling channels, but may damage thin walls if excessive pressure is applied.	316L
Shot Peening	Surface treatment by bombarding the material with small, hard particles to introduce compressive stresses.	Minimizes surface flaws and micro-cracks but can lead to uneven finishes on intricate geometries.	Roughness remains at around 5.9 µm, depending on intensity.	Improves wear resistance and fatigue performance by inducing beneficial compressive stresses.	Primarily used for external surfaces; not well-suited for internal or complex geometries.	316L
Laser Polishing	Using high-energy laser beams to melt and re-solidify surface layers for a smoother finish.	Eliminates surface asperities and reduces the prominence of peaks, though subsurface defects might remain.	Surface roughness decreases to around 2 µm.	Enhances surface hardness and wear resistance; improper use may lead to thermal damage.	Suitable for external surfaces; advanced robotic equipment may be required for complex shapes.	316/316L
Tumbling/Vibratory Finishing	Mass finishing method involving abrasive media in vibrating or rotating equipment to polish large batches.	Effectively removes surface imperfections on external parts; struggles with sharp edges and tight spaces.	Reduces surface roughness from 40 µm to around 4 µm.	Improves the overall surface finish and fit of components, rounding edges and eliminating burrs.	Time-consuming process (up to 96 h) with significant abrasive media waste.	316L
Ultrasonic Cavitation Abrasion	Uses ultrasonic waves in abrasive solutions to create cavitation bubbles that polish intricate surfaces.	Efficiently removes loose particles and surface imperfections, particularly in confined areas.	Reduces roughness by up to 20%, achieving around 3.8 µm.	Provides a polished finish without inducing stress or heat-related damage.	Ideal for internal features and small-scale geometries, such as micro-channels or narrow cavities.	316L

**Table 14:** Effect of shot peening on residual stresses and mechanical properties of reference and additively manufactured samples [168].

Sample Type	Sample Description	Mechanical Properties	Shot Peening Passes	Surface Residual Stress (MPa)	Residual Stress at 200 µm Depth (MPa)	Observations
Reference (REF) Sample chosen by author for comparison analysis	Conventional Manufactured	-(UTS): 1200 MPa -Yield Strength (YS): 900 MPa -Hardness: ~250 HV -Ductility: 20% Elongation	Un-peened	~0	~0	Baseline condition with no residual stress.
			1 Pass	−565	~0	Initial compressive stresses are induced at the surface.
			4 Passes	−657	~−300	Increased depth and magnitude of compressive stress.
			SSP (22 Passes)	−700	~−400	Significant compressive stresses, enhancing fatigue life.
Additively Manufactured (AM)	316L Stainless Steel (SLM)	-Ultimate Tensile Strength (UTS): 850 MPa -Yield Strength (YS): 650 MPa -Hardness: ~220 HV -Ductility: 30% Elongation	Un-peened	~+200 (Tensile)	~+100 (Tensile)	Tensile residual stresses were inherent in the as-built state.
			1 Pass	−550	~−250	Moderate compressive stress induced at the surface.
			4 Passes	−700	~−300	Substantial increase in compressive stresses.
			SSP (22 Passes)	−750	~−600	Deep compressive stresses with improved defect mitigation.

**Table 15:** Residual stress evolution under various heat treatment conditions for 316L stainless steel. Table reproduced with permission from publisher [25].

Heat Treatment Condition	Residual Stress (MPa)	Observations
As-printed	−248 ± 6	Significant compressive residual stress is present in the as-built condition.
400 °C × 4 h	−191.1 ± 14.5	Moderate stress relief of 24%, retaining compressive stress.
650 °C × 2 h	−90.5 ± 10.3	Stress relief of 65% was achieved, associated with partial dislocation annihilation.
1100 °C × 5 min	−18.9 ± 8.7	Nearly complete stress relief (~90%) after rapid cooling.
1100 °C × 30 min	Slightly reduced further	Minor further stress relief, with recrystallization and grain growth starting.
1400 °C × 10 min	Minimal compressive stress	Further grain coarsening and δ-ferrite formation lead to changes in microstructure and properties.

**Table 16:** Comparison of post-processing techniques (DED, SLM, EBM, wrought, cast) and their effects on material properties.

Process	Orientation	Conditions	YS (MPa)	US (MPa)	ε (%)	Ref.
DED	-	As-built	405–415	620–660	32–40	[191]
DED	-	1150 °C 2 h air-quenched	325–355	600–620	42–43	[191]
DED	-	As-built	-	720	56	[192]
DED	-	1060 °C 1 h vacuum-treated	-	605	78	[192]
DED	Horizontal	As-built	473.33 ± 4.10	665.00 ± 5.71	37.71 ± 1.63	[193]
DED	Vertical	As-built	387.00 ± 4.96	600.00 ± 4.54	36.67 ± 0.47	[193]
DED	Horizontal	Heat-treated	378.32 ± 3.57	632.67 ± 3.85	40.34 ± 1.49	[193]
DED	Vertical	Heat-treated	310.00 ± 4.54	566.67 ± 2.62	34.67 ± 3.29	[193]
SLM	Vertical	316L AF	427 ± 8	522 ± 5	15 ± 2	[25]
SLM	Horizontal	316L AF	406 ± 20	510 ± 4	18 ± 1	[25]
SLM	Horizontal	SLM-HIP densified 1150 °C × 3 h	201 ± 4	428 ± 13	38 ± 6	[25]
SLM	Vertical	316L AF	590 ± 17	705 ± 15	44 ± 7	[25]
SLM	-	SLM + solution annealing 1095 °C × 1 h	375 ± 11	635 ± 17	51 ± 3	[25]
SLM	Vertical	316L AF vertical	500	600.2 ± 2.2	55 ± 2.5	[25]
SLM	Vertical	SLM + stress-relief 650 °C × 2 h	475	617.9 ± 1.4	54.1 ± 1.6	[25]
SLM	Vertical	SLM + HIP densified 1150 °C × 3 h	375	586.6 ± 2.4	64.5 ± 2.9	[25]
SLM	Horizontal	SLM + stress–relief 388 °C × 4 h	496	717	28	[25]
SLM		SLM + solution annealing 1050 °C × 2 h	424 ± 8	673 ± 13	44 ± 3	[25]
SLM	-	SLM + solution annealing 1200 °C × 2 h	416 ± 9	684 ± 16	52 ± 3	[25]
SLM	-	As-printed	400.3 ± 3.1	572.8 ± 6.0	45.5 ± 0.3	[25]
SLM	-	400 °C × 4 h	418.3 ± 2.2	574.8 ± 0.7	45.8 ± 1.6	[25]
SLM	-	650 °C × 2 h	365.8 ± 2.0	550.5 ± 2.0	38.1 ± 0.9	[25]
SLM	-	800 °C × 2 h	327.3 ± 2.2	536.7 ± 5.5	32.5 ± 1.6	[25]
SLM	-	1100 °C × 5 min	311.9 ± 2.5	554.6 ± 4.6	57.5 ± 2.0	[25]
SLM	-	1100 °C × 30 min	307.8 ± 3.0	546.1 ± 2.3	51.5 ± 1.3	[25]
SLM	-	1100 °C × 8 h	293.5 ± 7.6	558.8 ± 2.3	50.5 ± 0.6	[25]
SLM	-	1400 °C × 10 min	232 ± 2.7	535.3 ± 2.2	43.3 ± 0.2	[25]
EBM	Horizontal	As built	334.2 ± 15.5	571.8 ± 19.3	29.3 ± 5.2	[97]
EBM	Horizontal	As built	342.9 ± 22.8	436.5 ± 23.2	9.6 ± 2.3	[97]
EBM	Vertical	As built	395.8 ± 9.0	651.7 ± 8.5	30.6 ± 3.0	[97]
EBM	Vertical	As built	315.7 ± 10.0	580.2 ± 6.8	35.2 ± 2.3	[97]
EBM	-	Preheat the build plate up to 820 °C Build temp (830–700 °C)	253 ± 3	509 ± 5	59 ± 3	[39]
EBM	-	(Test performed at ET of 250 °C)	152 ± 3	386 ± 3	46 ± 3	[39]
CAST	-	-	262	552	55	[153]
WROUGHT	-	-	170	480	40	[40]

**Table 17:** Comparison of microstructural features: SLM, DED, EBM (as built and post-processed).

Processing Conditions & Techniques	Grain Size (γ) (μm)	Aspect Ratio	Dislocation Cell Size (nm)	Inclusion Diameter (nm)	Phases Present	Microstructure Observations	Ref.
As-built (SLM)	14.8 ± 0.4	3.50 ± 0.07	466.4 ± 18.7	36.9 ± 1.3 (~0.37 vol%)	γ	-	[25]
400 °C × 4 h (SLM)	Not Changed	Not Changed	477.2 ± 20.3	Not Changed	γ	-	[25]
650 °C × 2 h (SLM)	Mostly Unchanged	Mostly Unchanged	505.4 ± 21.5	Mostly Unchanged	γ + σ	-	[25]
800 °C × 2 h (SLM)	-	-	535.6 ± 18.5	-	γ + ~0.11 vol% σ	-	[25]
1100 °C × 5 min (SLM)	14.6 ± 0.8	3.40 ± 0.07	Gradually Dispersed	52.6 ± 2.2	γ	-	[25]
1100 °C × 30 min (SLM)	17.9 ± 0.9	3.30 ± 0.07	Gradually Dispersed	54.8 ± 2.0	γ	-	[25]
1100 °C × 8 h (SLM)	33.1 ± 1.5	2.23 ± 0.03	Mostly Dispersed	87.9 ± 4.2 (~1.5 vol%)	γ + δ	-	[25]
1400 °C × 10 min (SLM)	32.7 ± 1.2	2.03 ± 0.03	Mostly Dispersed	Significant Coarsening	γ + ~13 vol% δ	-	[25]
As-built (DED)	87 ± 5	-	-	-	γ	Columnar grains observed, epitaxial grain growth, finer grains at the interface	[196]
650 °C for 2 h (annealed) HT1 (DED)	73 ± 3	-	-	-	γ + ferrite phase	Epitaxial grain growth; misplaced cell structures noticed	[196]
650 °C for 6 h (annealed) HT2 (DED)	65 ± 3	-	-	-	γ + ferrite phase	Higher epitaxial grain growth than HT1; misplaced cell structures noticed	[196]
(Annealed) 1150 °C for 2 h, HT3 (DED)	35 ± 2	-	-	-	γ + ferrite phase	epitaxial grain growth, equiaxed grains, Complete modification of cell structure; reduced yield strength	[196]
(Annealed) 1150 °C for 4 h + 1066 °C for 1 h, HT4 (DED)	22 ± 3	-	-	-	γ + ferrite phase	Equiaxed grains and coarsened grains compared to HT3 due to prolonged exposure.	[196]
(650 °C for 2 h and 650 °C for 6 h) Interface Area of PBF and DED where the substrate was PBF fabricated	Finer grains	-	-	-	γ + ferrite phase	Good metallurgical bond between DED and PBF; equiaxed grains dominate	[196]
DED annealed Zone area, 1150 °C for 4 h + 1066 °C for 1 h	Larger grains	-	-	-	γ	Grain coarsening was observed in the DED portion due to prolonged heat treatment.	[196]
PBF Zone for 1150 °C for 4 h + 1066 °C for 1 h (DED)	Coarse large grains	-	-	-	γ	Sluggish recrystallization kinetics observed due to particle coarsening	[196]
As-built (Hybrid additive/subtractive process) (DED)	-	-	-	-	γ Matrix + δ + σ	Strip morphology of δ- and σ-phases; uniform microstructure	[194]
950 °C/3 min/Water Quench (WQ) (DED)	-	-	-	-	γ + δ + σ	Vermicular morphology of δ- and σ-phases; partial spheroidization of σ-phase	[194]
1000 °C/3 min/WQ (DED)	-	-	-	-	γ + δ + σ	Slight reduction in the σ-phase within γ matrix	[194]
1050 °C/3 min/WQ (DED)	-	-	-	-	γ + δ	σ-phase replaced by γ matrix; δ-phase partially spheroidized	[194]
1150 °C/3 min/WQ (DED)	-	-	-	-	γ + δ	Σ-phase fully dissolved; δ-phase significantly decreased	[194]
1150 °C/30 min/WQ (DED)	-	-	-	-	γ	Fully austenitic microstructure achieved; grain coarsening observed	[194]
As-built (Fracture Surface) (DED)	-	-	-	-	-	Ductile fracture with fibrous appearance; small dimples with inhomogeneous distribution	[194]
1050 °C/3 min (Fracture Surface) (DED)	-	-	-	-	-	Deep dimples of large size; increased crack propagation resistance	[194]
820 °C Preheating of the build plate, Build Temp was (830–700 °C.) (EBM)	Upto 300 μm, with subgrains 1 and 9 μm	-	-	-	γ + δ	hierarchical microstructure, where pure austenite with less than 0.1% ferrite	[39]

## Data Availability

Not Applicable.

## Abbreviations

The following abbreviations are used in this paper:

AM	Additive Manufacturing
DED	Directed Energy Deposition
DEDo	Directed Energy Deposition only
DED + LS	Directed Energy Deposition with Laser Scanning
SLM	Selective Laser Melting
EBM	Electron Beam Melting
L-PBF/LPBF	Laser Powder Bed Fusion
HIP	Hot Isostatic Pressing
HT	Heat Treatment
HT1–HT4	Specific heat treatment stages (varied temperature)
SR	Stress Relief
SA	Solution Annealing
FM	Finish Machining
DF	Drag Finishing
VSF	Vibratory Surface Finishing
MAF	Magnetic Abrasive Finishing
Vf	Feed rate (in MAF process)
TLA	Three Letter Acronym
LD	Linear Dichroism
PBF	Powder Bed Fusion
FCC	Face-Centered Cubic
δ-ferrite	Delta Ferrite
σ-phase	Sigma Phase
γ	Austenite
EBSD	Electron Backscatter Diffraction
XRD	X-ray Diffraction
HV0.1	Vickers Hardness at 0.1 kgf
UTS	Ultimate Tensile Strength
YS	Yield Strength
R_a_	Arithmetic Average Roughness
R_z_	Maximum Profile Height
R_pv_	Peak-to-Valley Roughness
R_sk_	Roughness Skewness
R_ku_	Roughness Kurtosis
S_a_	Average Surface Roughness (area-based)
S_z_	Maximum Height of Surface Profile
S_dr_	Developed Surface Area Ratio
AISI	American Iron and Steel Institute
ASTM	American Society for Testing and Materials
Mo	Molybdenum
Cr	Chromium
Mn	Manganese
Ni	Nickel
Ti6Al4V	Titanium Alloy (Titanium–6% Aluminum–4% Vanadium)
AlSi10Mg	Aluminum-Silicon Alloy
IN718	Inconel 718
DLD	Direct Laser Deposition
REF	Reference Sample (conventionally manufactured)
SSP	Severe Shot Peening
LSF	Laser Solid Forming
sccm	Standard Cubic Centimeters per Minute (gas flow)
